# Obesity-associated reduction of miR-150-5p in extracellular vesicles promotes ventilator-induced lung injury by modulating the lysosomal degradation of VE-cadherin

**DOI:** 10.1038/s41420-025-02499-5

**Published:** 2025-05-06

**Authors:** Yi Zhang, Changping Gu, Liang Zhao, Bailun Wang, Yongtao Sun, Yalin Lou, Daqing Ma, Yuelan Wang

**Affiliations:** 1 Department of Anesthesiology, Shandong Provincial Hospital, Shandong University, Jinan, Shandong China; 2 Department of Anesthesiology, Shandong Provincial Hospital Affiliated to Shandong First Medical University, Jinan, Shandong China; 3 Department of Anesthesiology and Perioperative Medicine, The First Affiliated Hospital of Shandong First Medical University, Jinan, Shandong China; 4 Perioperative and Systems Medicine Laboratory and Department of Anesthesiology, Children’s Hospital, Zhejiang University School of Medicine, National Clinical Research Center for Child Health, Hangzhou, Zhejiang China; 5 Division of Anaesthetics, Pain Medicine and Intensive Care, Department of Surgery and Cancer, Faculty of Medicine, Imperial College London, Chelsea & Westminster Hospital, London, UK

**Keywords:** Respiratory distress syndrome, Mechanisms of disease

## Abstract

Obese patient has a high risk of ventilator-induced lung injury (VILI) but its underlying mechanisms remain elusive. This study was designed to explore the role of circulating plasma extracellular vesicles (EVs) on the progression of VILI in the context of obesity. After high tidal volume mechanical ventilation, mice treated with plasma EVs from obese patients developed more severe lung damage than mice treated with plasma EVs from normal controls. miRNA sequencing of plasma EVs from obese patients revealed a significant downregulation of miR-150-5p compared to the others. miR-150-5p was found to target on XBP1s which subsequently regulated RAB7 as verified through dual-luciferase assays. This pathway promoted lysosomal degradation of vascular endothelial (VE)-cadherin, leading to an increased endothelial permeability. Obese mice showed an enhanced XBP1s/RAB7 expression, reduced VE-cadherin levels, and aggravated endothelial barrier damage and all of which intensified VILI. Administration of miR-150-5p agomir in obese mice mitigated VILI. Thus, this study highlights the low levels of miR-150-5p in EVs from obese patients modulated VILI severity *via* the XBP1s/RAB7 axis and the lysosomal degradation of VE-cadherin.

## Introduction

The prevalence of obesity is significantly increasing and imposing a substantial burden on individuals’ health [1]. Obesity is a significant risk factor for the development of acute respiratory distress syndrome (ARDS) [2], for which primary treatment is mechanical ventilation [3]. However, mechanical ventilation may also lead to lung injury, namely ventilator-induced lung injury (VILI) [4], which contributes to high morbidity and mortality in ventilated patients particularly who are obese. Obesity has been reported to promote a pro-inflammatory state and exacerbate lung ischemia-reperfusion injury [5]. Obesity also increases lung injury under PM_2.5_ exposure [6]. All these may be associated with an increased inflammatory response, oxidative stress and cellular senescence, and impaired tissue repair mechanisms. However, the impact of obesity on the development of the pathological process of acute lung injury (ALI)/ARDS remains inconclusive.

The pathogenesis of VILI is characterized by the increased permeability of the alveolar capillary membrane, which is closely associated with the disruption of the pulmonary vascular endothelial (VE) barrier [7]. The barrier is primarily governed by adherent junctions and tight junctions. Among the components of the adherent junction complex, VE-cadherin plays a crucial role in maintaining vascular integrity [8]. VE-cadherin is regulated through endocytosis, and the endocytosed VE-cadherin is processed via the endosome-lysosome pathway. Inhibition of lysosomes prevents VE-cadherin degradation [9]. Disruption of the VE-cadherin-catenin complex triggers breakdown of adherent junctions between endothelial cells, causing endothelial barrier dysfunction and increased permeability [10]. However, research directly linking VILI-induced endothelial barrier dysfunction and VE-cadherin’s endocytosis and lysosomal degradation is limited and its influence in obese patients on these processes remains unexplored.

Extracellular vesicles (EVs) are key players in the transmission of biological signals between cells [11]. They modulate cellular processes through releasing active substances, including miRNAs that regulate various pathological processes [12, 13]. Plasma EVs derived from septic patients are shown to exacerbate various phenotypes related to ALI through impacting autophagy, inflammatory response, apoptosis and endothelial barrier permeability [14]. Furthermore, plasma EVs from trauma/hemorrhagic shock patients are found to increase lung permeability and histopathological damage [15]. These findings imply that EVs derived from various patient populations impact lung injury outcomes.

The objective of this study was to investigate the impact of plasma EVs derived from obese patients on VILI through examining the potential effects of miR-150-5p in EVs on VILI and underlying mechanisms.

## Results

### Effects of plasma EVs from obese patients on VILI in mice

**Fig. 1:**
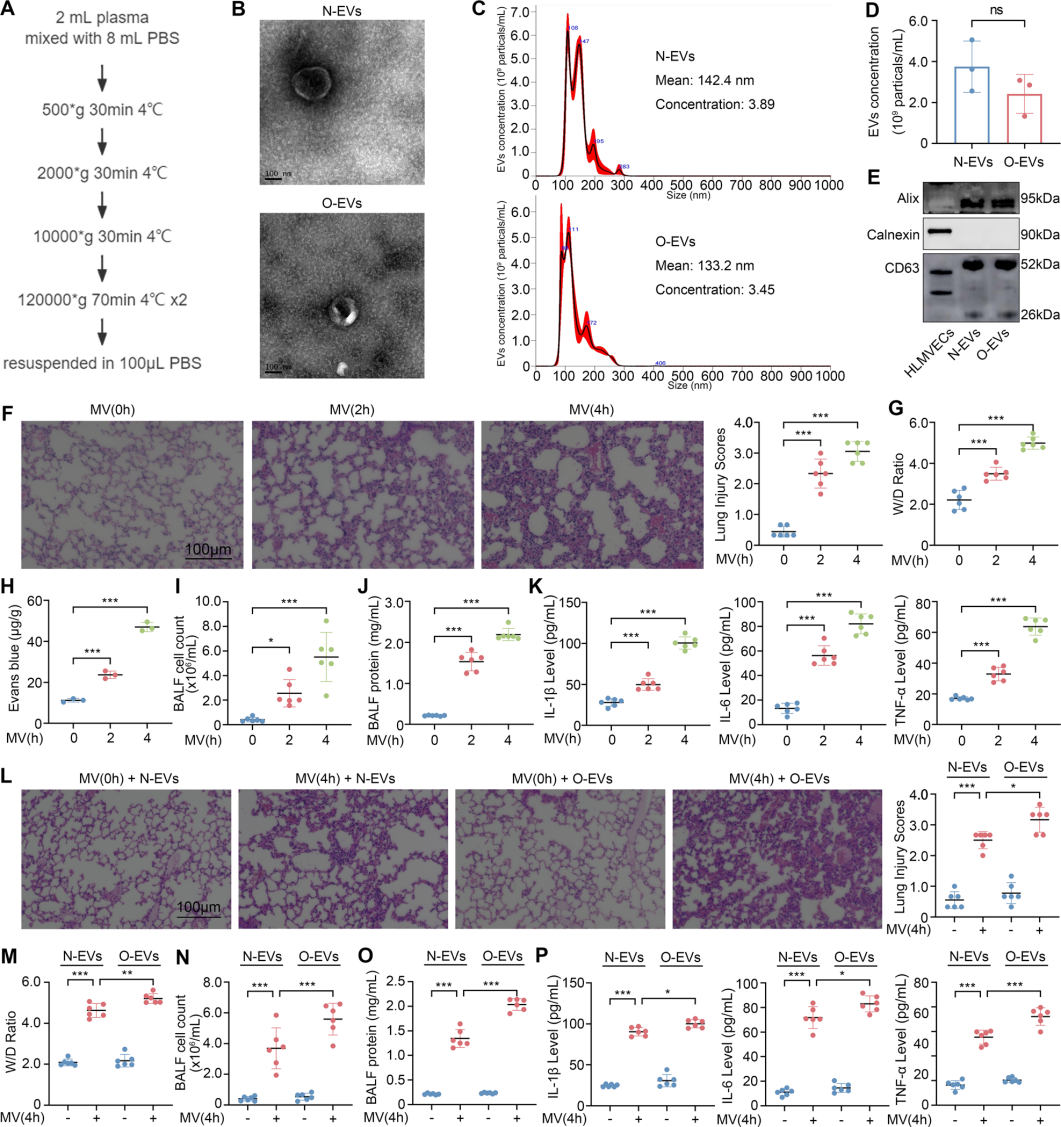
Effects of plasma EVs from obese patients on VILI in mice. **A** The process of plasma EVs extraction using differential centrifugation. **B** Representative TEM images of EVs from normal controls and obese patients. **C, D** Measurement of EVs from normal controls and obese patients’particle size and concentration using NTA (*n* = 3). **E** EVs representative markers detected via western blot. **F** Lung injury scores assessed by HE staining of the lung injury of mice subjected to mechanical ventilation for 0, 2, 4 h (*n* = 6). **G** Lung W/D ratio of mice subjected to mechanical ventilation for 0, 2, 4 h (**n** = 6). **H** Lung Evans blue dye leakage of mice subjected to mechanical ventilation for 0, 2, 4 h (*n* = 3). **I** BALF cell count of mice subjected to mechanical ventilation for 0, 2, 4 h (*n* = 6). **J** BALF protein concentration of mice subjected to mechanical ventilation for 0, 2, 4 h (*n* = 6). **K** The levels of IL-1β, IL-6, TNF-α in BALF of mice subjected to mechanical ventilation for 0, 2, 4 h (*n* = 6). **L** Lung injury scores of mice after treatment with EVs from normal controls or obese patients followed by 4-h mechanical ventilation (*n* = 6). **M** Lung W/D ratio of mice after treatment with EVs from normal controls or obese patients followed by 4-h mechanical ventilation (*n* = 6). **N** BALF cell count of mice after treatment with EVs from normal controls or obese patients followed by 4-h mechanical ventilation (*n* = 6). **O** BALF protein concentration of mice after treatment with EVs from normal controls or obese patients followed by 4-h mechanical ventilation (*n* = 6). **P** The levels of IL-1β, IL-6, TNF-α in BALF of mice after treatment with EVs from normal controls or obese patients followed by 4-h mechanical ventilation (*n* = 6). Data are expressed as the mean ± SD; *P < 0.05, **P < 0.01, ***P < 0.001, ns not significant, N-EVs EVs from normal controls, O-EVs EVs from obese patients, MV mechanical ventilation.

EVs were isolated from the plasma of normal controls and obese patients through differential centrifugation (Fig. 1A). EVs from both groups exhibited typical round and cup-shaped morphologies with an average diameter of 50–150 nm, and Apolipoprotein A1, which represents lipoprotein contamination, showed no difference in expression between the two EVs (Figs. 1B–D and S1A, B). Both the EVs expressed EVs-specific markers Alix and CD63, but not the negative marker Calnexin (Fig. 1E). The EVs from both groups were no significant differences in regarding their characteristics.

We examined the establishment of mouse model of VILI. Mechanical ventilation triggered extensive lung damage characterized by edema, atelectasis, necrosis, inflammation, hemorrhage, and hyaline membrane formation. Correspondingly, lung injury scores (Fig. 1F, *P* < 0.001), lung Wet/dry (W/D) weight ratio (*P* < 0.001, Fig. 1G), lung Evans blue dye leakage(*P* < 0.001, Fig. 1H), bronchoalveolar lavage fluid (BALF) cell count (*P* < 0.001, Fig. 1I), BALF protein concentration (*P* < 0.001, Fig. 1J), and BALF concentrations of IL-1β, IL-6, TNF-α (*P* < 0.001, *P* < 0.001, *P* < 0.001, respectively, Fig. 1K) were significantly increased following 4-h mechanical ventilation. Collectively, these data confirmed that high tidal volume of mechanical ventilation induced inflammation and histopathological damage.

Then we injected mice with EVs from normal controls or obese patients through the tail vein and treated them with 4-h mechanical ventilation. The severe lung injury was found to be significant in mice injected with EVs from obese patients compared with those injected with EVs from normal controls as measured with lung injury scores(*P* < 0.05, Fig. 1L), lung W/D weight ratio (*P* < 0.01, Fig. 1M), BALF cell count (*P* < 0.001, Fig. 1N), BALF protein concentration (*P* < 0.001, Fig. 1O), and BALF concentrations of IL-1β, IL-6, TNF-α (*P* < 0.05, *P* < 0.05, *P* < 0.001, respectively, Fig. 1P).

### Cyclic stretch induces the lysosomal degradation of VE-cadherin

**Fig. 2:**
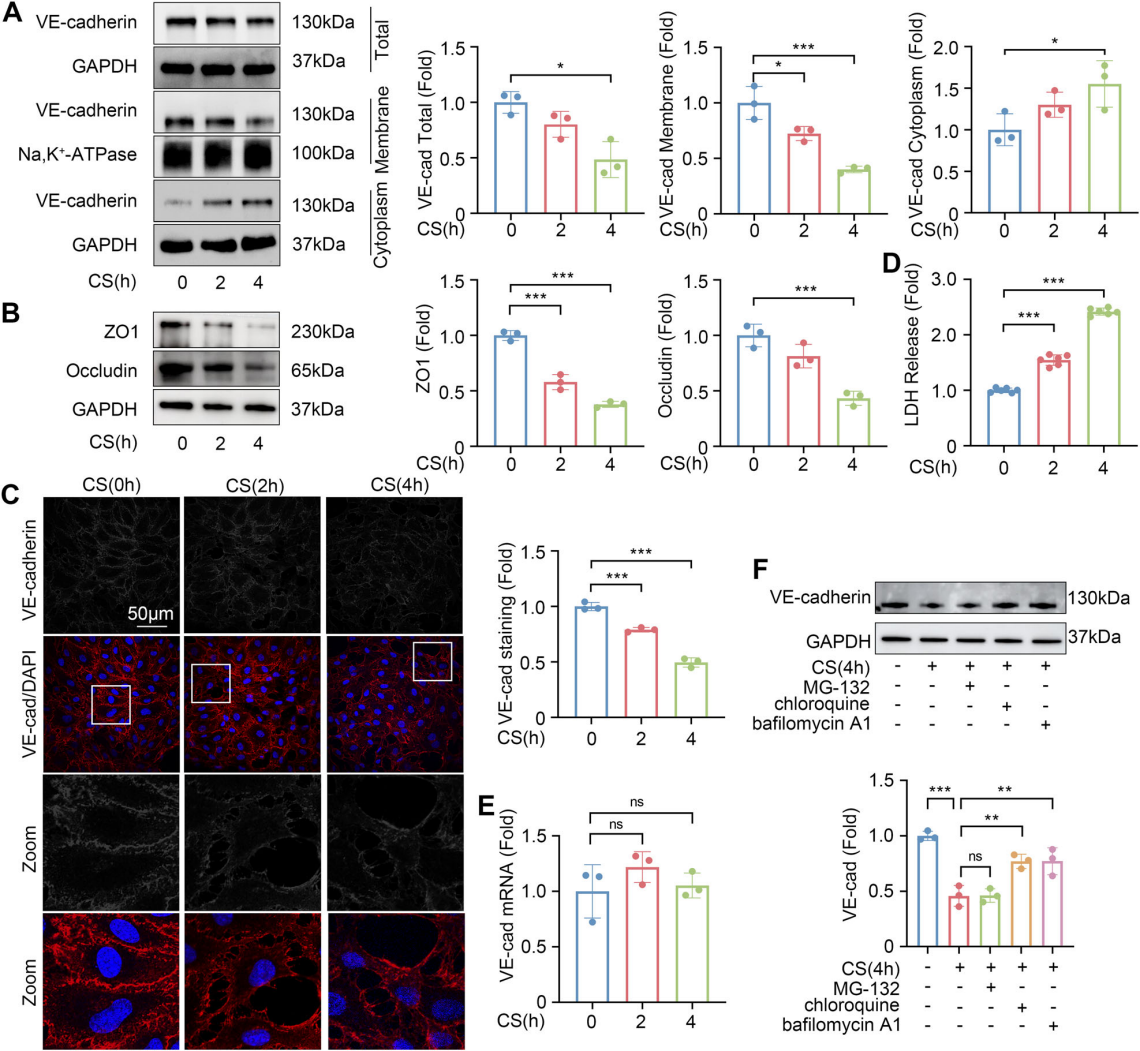
Cyclic stretch induces the lysosomal degradation of VE-cadherin and endothelial barrier disruption in HLMVECs. **A** Western blot analysis of VE-cadherin expression levels in HLMVECs across different cellular compartments after cyclic stretch for 0, 2, 4 h (*n* = 3). **B** Western blot analysis of ZO1 and Occludin expression levels in HLMVECs after cyclic stretch for 0, 2, 4 h (*n* = 3). **C** Representative confocal images (shown both in gray and color scale) and quantitative analysis of VE-cadherin staining in HLMVECs after cyclic stretch for 0, 2, 4 h (*n* = 3). **D** LDH release ratio in HLMVECs after cyclic stretch for 0, 2, 4 h (*n* = 6). **E** Quantitative analysis of VE-cadherin mRNA expression levels in HLMVECs after cyclic stretch for 0, 2, 4 h (*n* = 3). **F** Western blot analysis of VE-cadherin in HLMVECs after treatment with proteasome inhibitor or lysosome inhibitor followed by 4-h cyclic stretch (*n* = 3). Data are presented as the mean ± SD; *P < 0.05, **P < 0.01, ***P < 0.001, ns not significant, CS cyclic stretch, N-EVs EVs from normal controls, O-EVs EVs from obese patients.

To explore the mechanism by which EVs from different sources function, we treated human lung microvascular endothelial cells (HLMVECs) with cyclic stretch to simulate mechanical ventilation in vitro. VE-cadherin, a crucial component of HLMVECs junctions, was predominantly localized at the cell membrane under normal condition (Figs. 2A and S2A). Prolonged cyclic stretch exposure progressively diminished membrane-bound VE-cadherin, with its expression on the cell membrane decreased to less than 50% after 4-h cyclic stretch compared to the untreated cells (*P* < 0.001). Simultaneously, the level of cytoplasmic VE-cadherin showed ~1.5-fold increase (*P* < 0.05), suggesting its endocytosis. Additionally, cyclic stretch also resulted in a reduction in the total amount of VE-cadherin and tight junction protein ZO1 and Occludin (Fig. 2B).

We further analyzed the cell injury induced by cyclic stretch using confocal microscopy (Fig. 2C). Under control condition, VE-cadherin formed a continuous line at the HLMVECs border, indicative of intact cell-cell junctions. However, with increasing cyclic stretch exposure, VE-cadherin staining intensity decreased, and VE-cadherin staining became interrupted, intercellular gaps emerged, suggesting cyclic stretch induced VE-cadherin disassembly and subsequent cytoskeletal remodeling that disrupts the integrity of the endothelial barrier. Lactate dehydrogenase (LDH) is an important indicator of membrane damage [16]. The increase in LDH release after cyclic stretch also reflected the damage of the cell membrane (Fig. 2D).

Cyclic stretch did not change the mRNA levels of VE-cadherin (Fig. 2E) but the lysosome inhibitor chloroquine and bafilomycin A1 pretreating HLMVECs blocked the decrease of VE-cadherin (Figs. 2F and S2B), indicating that cyclic stretch induced endocytosis of VE-cadherin, then leading to lysosomal degradation.

### miR-150-5p is important for cellular barrier function

**Fig. 3:**
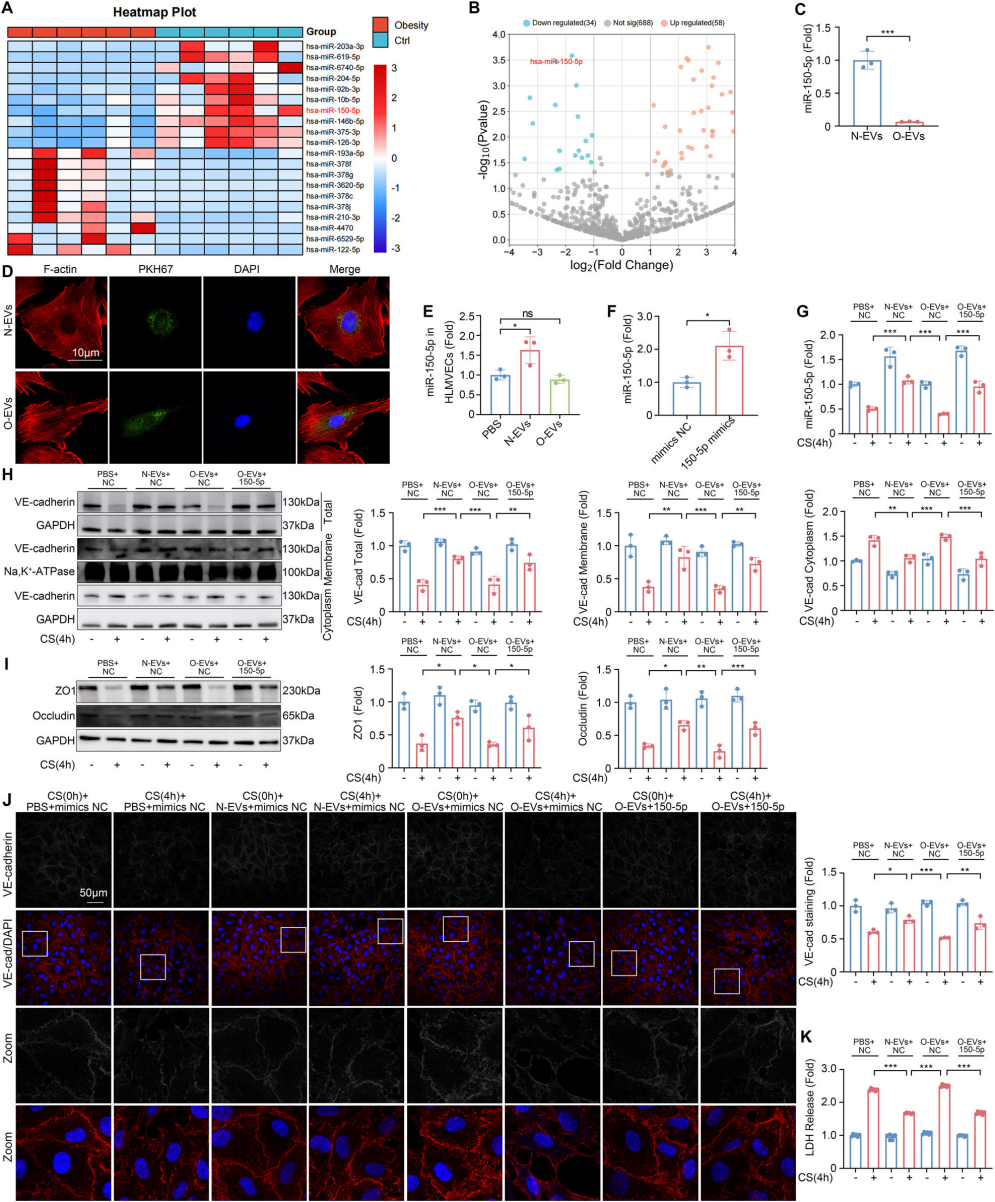
miR-150-5p regulates VE-cadherin. **A** Comparison of DE-miRNAs in EVs from normal controls and obese patients. **B** Volcano plot illustrating the EVs miRNAs detected by microarrays. **C** miR-150-5p expression levels in EVs from normal controls and obese patients (*n* = 3). **D** Confocal images of HLMVECs incubated with PKH67-labeled EVs. **E** Quantitative analysis of miR-150-5p expression levels in HLMVECs incubated with EVs from normal controls or EVs from obese patients (*n* = 3). **F** Verification of miR-150-5-p mimics efficiency (*n* = 3). **G** Quantitative analysis of miR-150-5p expression levels in HLMVECs of different groups (*n* = 3). **H** Western blot analysis of VE-cadherin in HLMVECs across different cellular compartments after different treatments (*n* = 3). **I** Western blot analysis of ZO1 and Occludin expression levels in HLMVECs of different groups (*n* = 3). **J** Representative confocal images (shown both in gray and color scale) and quantitative analysis of VE-cadherin staining in HLMVECs of different groups (*n* = 3). **K** LDH release ratio in HLMVECs of different groups (*n* = 6). Data are expressed as the mean ± SD; *P < 0.05, **P < 0.01, ***P < 0.001, ns not significant, CS cyclic stretch, N-EVs EVs from normal controls, O-EVs EVs from obese patients.

To investigate the underlying mechanism of EVs from obese patients in exacerbating VILI, we conducted miRNA microarray sequencing on EVs from normal controls and obese patients. A total of 92 known differential expressed miRNAs (DE-miRNAs) were identified, with 58 up-regulated and 34 down-regulated (Fig. 3A, B). Among these, miR-150-5p emerged as particularly significant due to its known role in inflammation regulation, oxidative stress, and ferroptosis in various diseases [17–19]. Its reduction has been previously shown in the progression of ARDS and other pulmonary conditions [20–23].

miR-150-5p levels were significantly lower in the EVs from obese patients compared to that from normal controls (*P* < 0.001, Fig. 3C). To explore the effects of EVs from both groups on HLMVECs, HLMVECs were cultured with plasma EVs from normal controls or obese patients. Confocal microscopy revealed the presence of labeled EVs in the cytoplasm of the recipient cells, indicating the effective uptake of EVs from both groups (Fig. 3D). miR-150-5p in HLMVECs was significantly increased after co-incubated with EVs from normal controls, while EVs from obese patients had no effect (Fig. 3E). Cyclic stretch reduced miR-150-5p expression, co-incubation with EVs from normal controls but not EVs from obese patients increased the content of miR-150-5p in HLMVECs (Fig. 3F, G) and alleviated the increased lysosomal degradation of VE-cadherin and the downregulation of ZO1 and Occludin caused by cyclic stretch (Fig. 3H, I). miR-150-5p mimics increased the content of miR-150-5p in HLMVECs co-incubated with EVs from obese patients and exerted a therapeutic effect on VE-cadherin, ZO1 and Occludin (Fig. 3G–I). Compared with EVs from normal controls, EVs from obese patients had no effect on cell membrane integrity as measured with immunofluorescent staining and LDH release (Fig. 3J, K).

### miR-150-5p regulates XBP1s and RAB7

**Fig. 4:**
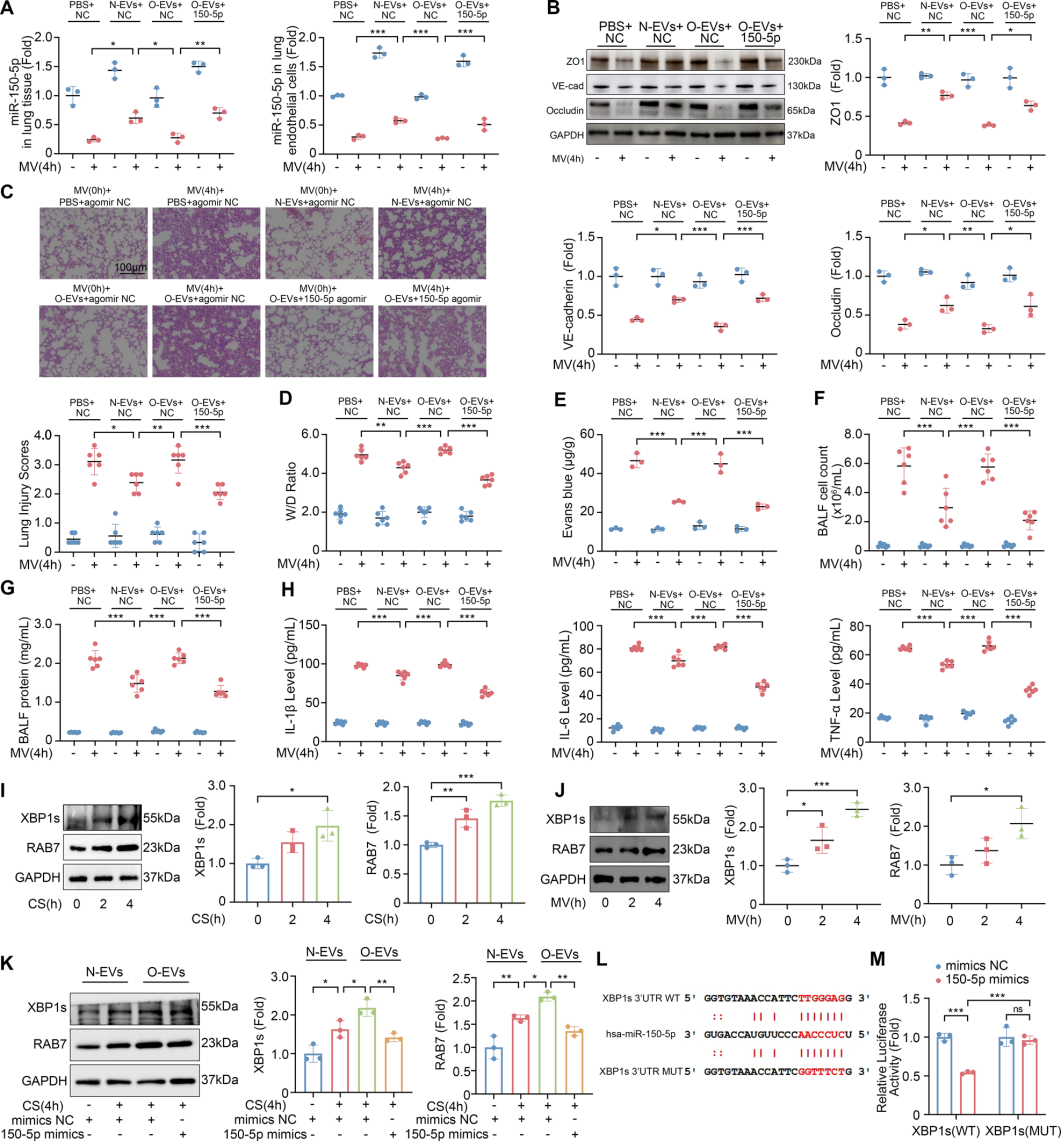
miR-150-5p alleviates the upregulation of XBP1 and RAB7 caused by EVs from obese patients. **A** Quantitative analysis of miR-150-5p expression levels in lung tissues and lung endothelium of different groups of mice (*n* = 3). **B** Western blot analysis of VE-cadherin, ZO1, and Occludin expression levels in lung tissues of different groups of mice (*n* = 3). **C** Lung injury scores of mice of different groups (*n* = 6). **D** Lung W/D ratio of mice of different groups (*n* = 6). **E** Lung Evans blue dye leakage of mice of different groups (*n* = 3). **F** BALF cell count of mice of different groups (*n* = 6). **G** BALF protein concentration of mice of different groups (*n* = 6). **H** The levels of IL-1β, IL-6, TNF-α in BALF of mice of different groups (*n* = 6). **I** Western blot analysis of XBP1s and RAB7 in HLMVECs after cyclic stretch for 0, 2, 4 h (*n* = 3). **J** Western blot analysis of XBP1s and RAB7 in mice subjected to mechanical ventilation for 0, 2, 4 h (*n* = 3). **K** Western blot analysis of XBP1s and RAB7 in HLMVECs transfected with miR-150-5p mimics followed by 4-h cyclic stretch (*n* = 3). **L** The predicted binding site between miR-150-5p and XBP1s. **M** Dual luciferase reporter assay confirmation of the relationship between miR-150–5p and XBP1s (*n* = 3). Data are presented as the mean ± SD; *P < 0.05, **P < 0.01, ***P < 0.001, ns not significant, CS cyclic stretch, N-EVs EVs from normal controls, O-EVs EVs from obese patients, MV mechanical ventilation.

Mechanical ventilation caused a decrease of miR-150-5p in lung tissues and lung endothelium of VILI mice, while EVs from normal controls increased miR-150-5p in lung tissues and lung endothelium (Fig. 4A). After mechanical ventilation, VE-cadherin, ZO1 and Occludin expression levels in mice injected with EVs from normal controls were increased (Fig. 4B), and lung injury was attenuated (Fig. 4C–H). This was not the case after injection with EVs from obese patients. However, 150-5p agomir treatment improved the lung injury of mice injected with EVs derived from obese patients (Fig. 4A–H).

To demonstrate the lung protective effect of miR-150-5p in circulating EVs, we created a mouse model with low plasma expression of miR-150-5p through miR-150-5p antagomir (Fig. S3A), and then extracted mouse plasma EVs (Fig. S3B). The miR-150-5p in the plasma EVs of mice injected with miR-150-5p antagomir was significantly reduced (Fig. S3C). The plasma EVs from normal control mice administered to VILI mice increased miR-150-5p (Fig. S3D) and VE-cadherin, ZO1, and Occludin in the lung tissues (Fig. S3E), and also alleviated lung injury (Fig. S3F–K). However, the plasma EVs of mice before given miR-150-5p antagomir did not show therapeutic effects(Fig. S3D–K).

We then explored the target genes of miR-150-5p, databases like miRDB, miDIP, and starBase were used to predict the potential target gene of miR-150-5p. Notably, X-box-binding protein 1s (XBP1s), a gene associated with cell barrier function, was identified as a potential target [24]. Gene Ontology (GO) and Kyoto Encyclopedia of Genes and Genomes (KEGG) enrichment analysis of DE-miRNAs highlighted their involvement in key cellular components or functions such as cell adhesion molecule binding, extrinsic component of membrane, and regulation of vesicle-mediated transport (Fig. S3L, M). The enrichment of DE-miRNAs in pathways related to small GTPase-mediated signal transduction, Ras protein signaling, and lysosome was considered to be Ras-related protein RAB7, a regulator of lysosomal function [25].

Concurrently, the expression of XBP1s and RAB7 was also induced in response to cyclic stretch and mechanical ventilation(Fig. 4I, J). When HLMVECs co-cultured with EVs from obese patients were subjected to cyclic stretch, the upregulation of XBP1s and RAB7 was more higher than HLMVECs co-cultured with EVs from normal controls, but this upregulation was mitigated in the presence of miR-150-5p mimics (Fig. 4K). To confirm the regulatory effects of miR-150-5p on XBP1s and RAB7, we performed dual luciferase reporter assay and found that overexpression of miR-150-5p damaged the activity of XBP1s-3′UTR-WT reporter (*P* < 0.001, Fig. 4L, M), but there was no significant change in the activity of MUT reporter, verifying the regulatory effect of miR-150-5p on XBP1s.

### Knockdown of XBP1s affects RAB7 and VE-cadherin expression

**Fig. 5:**
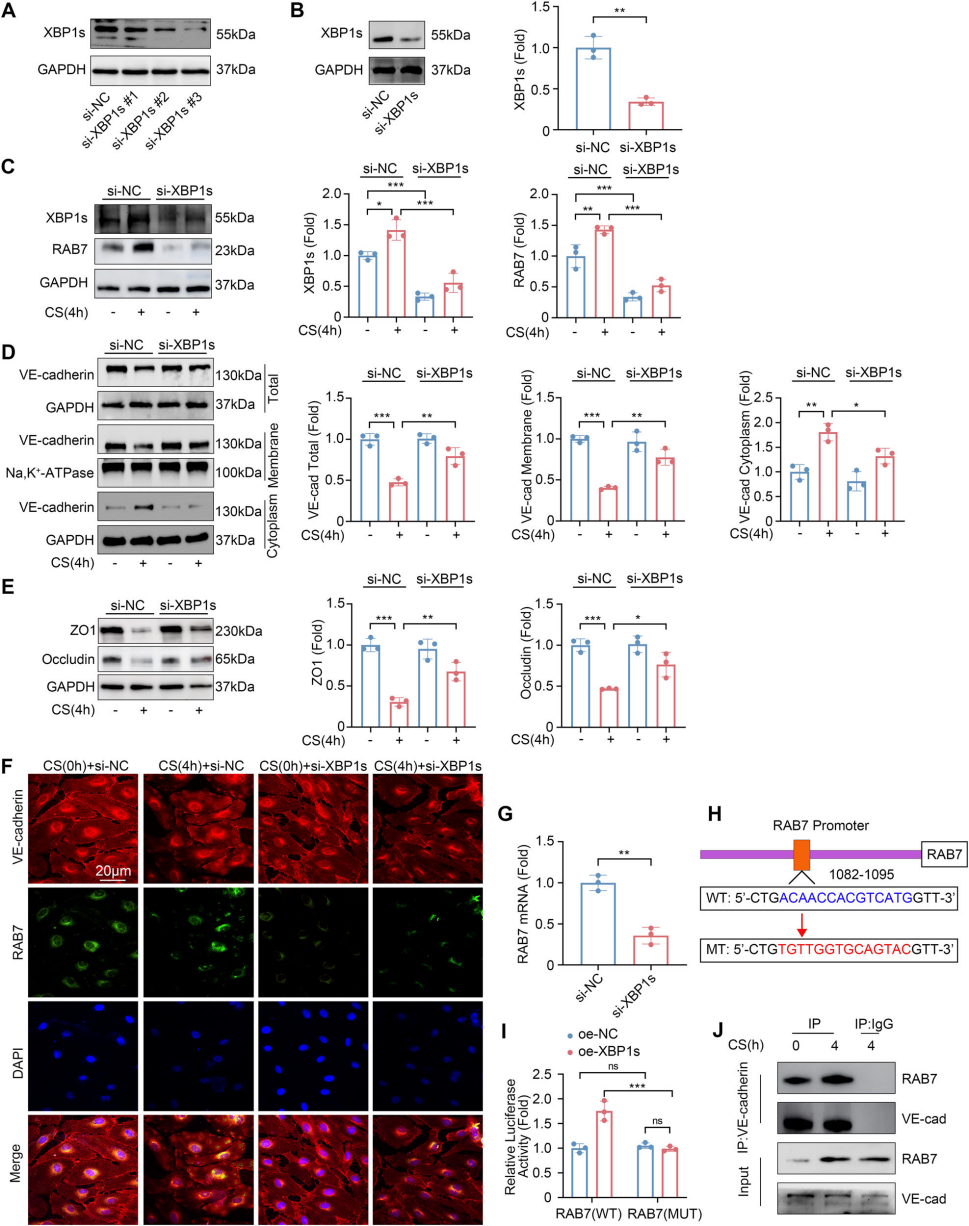
XBP1s transcriptionally regulates RAB7. **A**, **B** Western blot analysis of XBP1s in HLMVECs transfected with XBP1s siRNA or control siRNA (*n* = 3). **C** Western blot analysis of XBP1s and RAB7 in HLMVECs transfected with XBP1s siRNA followed by 4-h cyclic stretch (*n* = 3). **D** Western blot analysis of VE-cadherin in HLMVECs across different cellular compartments after transfection with XBP1s siRNA followed by 4-h cyclic stretch (*n* = 3). **E** Western blot analysis of ZO1 and Occludin expression levels in HLMVECs transfected with XBP1s siRNA (*n* = 3). **F** Representative confocal images showing the distribution and expression of VE-cadherin and RAB7. **G** Quantitative analysis of RAB7 mRNA expression level in HLMVECs transfected with XBP1s siRNA (*n* = 3). **H** Constructs of WT and MUT RAB7 promoter. **I** Luciferase activity determined in HEK 293T cells transfected with WT or MUT RAB7 luciferase reporter plasmids (*n* = 3). **J** Detection of the interaction between RAB7 and VE-cadherin in HLMVECs. Data are expressed as the mean ± SD; *P < 0.05, **P < 0.01, ***P < 0.001, ns not significant, CS cyclic stretch, IP immunoprecipitation.

To investigate the role of XBP1s in endothelial barrier function, HLMVECs were transfected with XBP1s small interfering RNA (siRNA), and the si-XBP1s #3 was used for subsequent experiments (Fig. 5A, B). XBP1s siRNA significantly mitigated the cyclic stretch-induced upregulation of RAB7 (Fig. 5C). After cyclic stretch, compared with those transfected with control siRNA, the total VE-cadherin and membrane VE-cadherin were increased, and the cytoplasmic VE-cadherin was decreased in HLMVECs transfected with XBP1s siRNA (*P* < 0.01, *P* < 0.01, *P* < 0.05, respectively, Fig. 5D). The use of XBP1s siRNA also rescued the decreased expression levels of ZO1 and Occludin induced by cyclic stretch (*P* < 0.01, *P* < 0.05, respectively, Fig. 5E). Confocal microscopy further illustrated the inverse correlation between VE-cadherin and RAB7 expression and distribution patterns (Fig. 5F). The XBP1s siRNA treatment resulted in an increase in VE-cadherin expression and a concurrent decrease in RAB7 expression, emphasizing the regulatory role of XBP1s in these pathways.

XBP1s siRNA transfection induced a reduction in RAB7 mRNA levels (*P* < 0.01, Fig. 5G). Using the Jaspar database, RAB7 was identified as a potential target gene for the XBP1s transcription factor. To test this hypothesis, we constructed luciferase vectors with both RAB7A-WT and RAB7A-MUT promoters fused with luciferase gene, as well as XBP1s overexpression plasmid for dual-luciferase reporter assays (Fig. 5H). We found a significant upregulation of transcriptional activity in XBP1s overexpression and the RAB7-WT promoter co-transfection HEK 293T cells. Mutation of the binding site in the RAB7-MUT promoter did not respond to XBP1s overexpression-induced transcriptional activity (Fig. 5I). Additionally, Co-immunoprecipitation (Co-IP) highlighted an increasing association between RAB7 and VE-cadherin under cyclic stretch, suggesting that XBP1s might regulate VE-cadherin through RAB7 (Fig. 5J).

### RAB7 mediates XBP1s-induced VE-cadherin lysosomal degradation and endothelial dysfunction

**Fig. 6:**
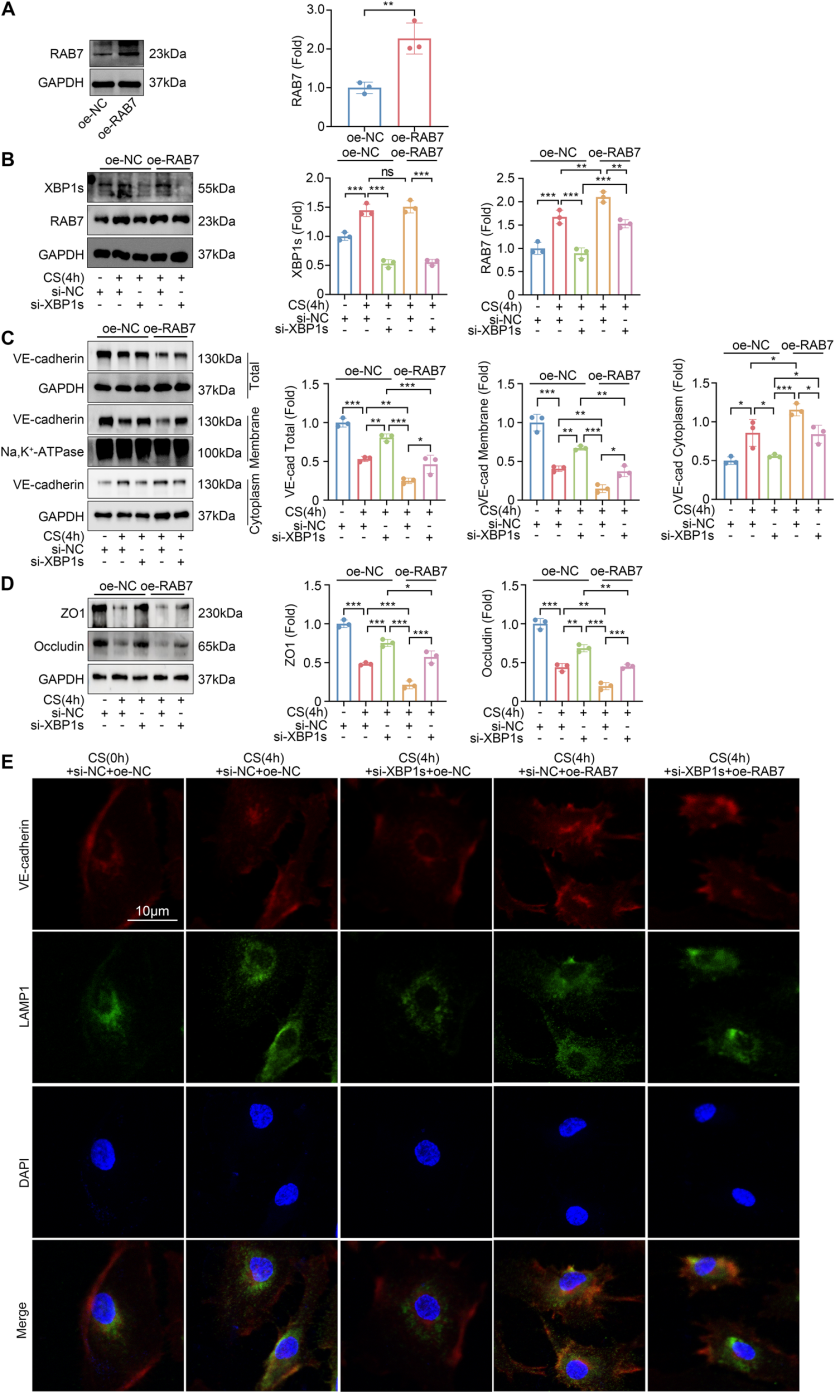
XBP1s regulates VE-cadherin by targeting RAB7. **A** Western blot analysis of RAB7 in HLMVECs transfected with RAB7 expression plasmids or control vector (*n* = 3). **B** Western blot analysis of XBP1s and RAB7 in HLMVECs transfected with XBP1s siRNA or RAB7 expression plasmids then cyclic stretch-treated for 4 h (*n* = 3). **C** Western blot analysis of VE-cadherin in HLMVECs across different cellular compartments after transfection with XBP1s siRNA or RAB7 overexpression plasmid followed by 4-h cyclic stretch (*n* = 3). **D** Western blot analysis of ZO1 and Occludin expression levels in HLMVECs after transfection with XBP1s siRNA or RAB7 overexpression plasmid followed by 4-h cyclic stretch (*n* = 3). **E** Representative confocal images showing the co-localization of VE-cadherin and LAMP1 in the cytoplasm. Data are expressed as the mean ± SD; *P < 0.05, **P < 0.01, ***P < 0.001, ns not significant, CS cyclic stretch.

Our present data indicate that XBP1s might affect the lysosomal degradation of VE-cadherin by regulating the transcription of RAB7. To gain further insights into the impact of RAB7 on VE-cadherin loss triggered by cyclic stretch and XBP1s, we overexpressed RAB7 (Fig. 6A) in HLMVECs and followed by cyclic stretch treatment.

RAB7 overexpression did not affect XBP1s levels (*P* > 0.05, Fig. 6B), reinforcing the notion that RAB7 acts downstream of XBP1s. Interestingly, RAB7 overexpression mitigated the protective impact of XBP1s siRNA on HLMVECs (Total VE-cadherin down-regulated by 54%, membrane VE-cadherin down-regulated by 63%, and cytoplasmic VE-cadherin up-regulated by 1.7-fold), which was in contrast to the effect of XBP1s siRNA (Fig. 6C). The protective effect of XBP1s siRNA on ZO1 and Occludin in HLMVECs was also reversed by RAB7 overexpression (Fig. 6D).

HLMVECs subjected to cyclic stretch showed an increased co-localization of VE-cadherin with lysosomal associated membrane protein 1 (LAMP1) in the cytoplasm. However, this co-localization was reduced when cells were transfected with XBP1s siRNA. In contrast, the combined transfection of RAB7 overexpression plasmid and XBP1s siRNA negated this protective effect. Notably, cells transfected solely with the RAB7 overexpression plasmid exhibited the most pronounced VE-cadherin loss post-cyclic stretch (Fig. 6E). The results collectively demonstrated that RAB7 played a crucial role in regulating the expression and distribution of VE-cadherin in HLMVECs induced by cyclic stretch and XBP1s.

### miR-150-5p mitigates VILI in obese mice

**Fig. 7:**
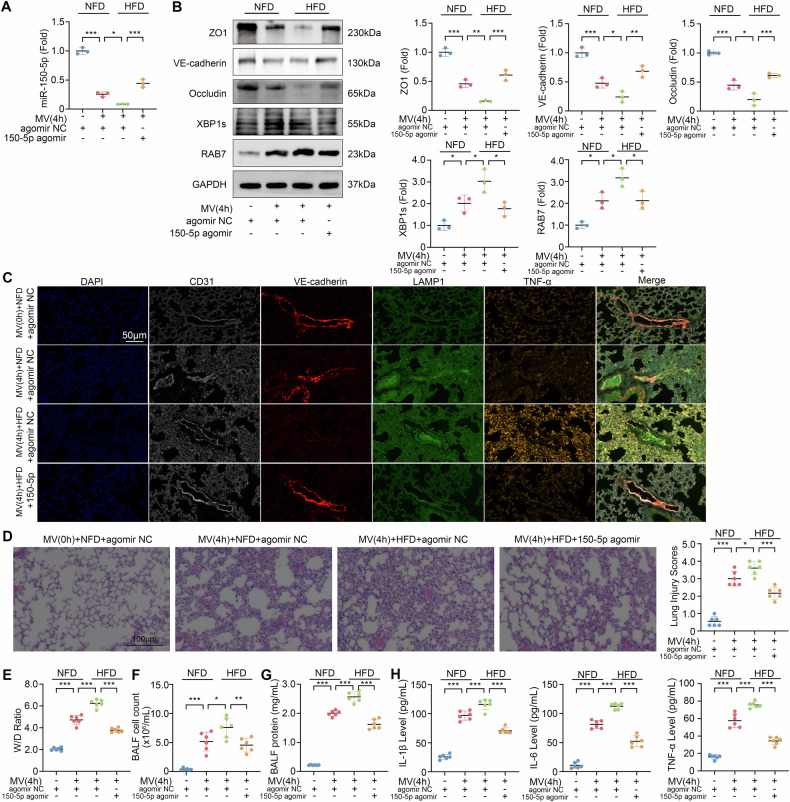
Treatment of miR-150-5p agomir alleviates VILI. **A** Quantitative analysis of miR-150-5p expression levels in lung tissues of NFD-fed mice and HFD-fed mice (*n* = 3). **B** Western blot analysis of VE-cadherin, ZO1, Occudin, XBP1s, and RAB7 in NFD-fed mice and HFD-fed mice subjected to 4-h mechanical ventilation (*n* = 3). **C** The expression levels of endothelial markers CD31, VE-cadherin, LAMP1, and TNF-α in lung tissue were observed by multiplex IHC. **D** Lung injury scores of NFD-fed mice and HFD-fed mice after treatment with miR-150-5p agomir followed by 4-h mechanical ventilation *n* = 6. **E** Lung W/D ratio of NFD-fed mice and HFD-fed mice after treatment with miR-150-5p agomir followed by 4-h mechanical ventilation (*n* = 6). **F** BALF cell count of NFD-fed mice and HFD-fed mice after treatment with miR-150-5p agomir followed by 4-h mechanical ventilation *n* = 6. **G** BALF protein concentration of NFD-fed mice and HFD-fed mice after treatment with miR-150-5p agomir followed by 4-h mechanical ventilation (*n* = 6). **H** The levels of IL-1β, IL-6, TNF-α in BALF of NFD-fed mice and HFD-fed mice after treatment with miR-150-5p agomir followed by 4-h mechanical ventilation (*n* = 6). Data are expressed as the mean ± SD; *P < 0.05, **P < 0.01, ***P < 0.001, ns not significant, MV mechanical ventilation, NFD normal fat diet, HFD high fat diet.

The above in vitro data demonstrated that miR-150-5p in EVs is important for cellular barrier function. We hypothesize that administration of miR-150-5p may protect from VILI in obese condition. We first induced obesity in mice with a high-fat diet (HFD) and administered miR-150-5p agomir intranasally before subjected them to high tidal volume mechanical ventilation (Fig. 7A). Compared with the normal fat diet (NFD)-fed mice without mechanical ventilation, miR-150-5p expression in both the NFD-fed mice and HFD-fed mice with mechanical ventilation was reduced by 74.4% and 91.4%, respectively. In the HFD-fed mice with mechanical ventilation treated with 150-5p agomir, the expression level of 150-5p in the lung tissues increased to 44.2% of that in the NFD-fed mice without mechanical ventilation.

The upregulation of XBP1s and RAB7 and loss of VE-cadherin, ZO1, and Occludin in the HFD-fed mice after mechanical ventilation treatment (24% of VE-cadherin expression) were more severe than that of NFD-fed mice (48% of VE-cadherin expression). Intriguingly, miR-150-5p agomir treatment suppressed the increase in XBP1s and RAB7 expression and mitigated the loss of VE-cadherin, ZO1, and Occludin in HFD-fed mice (Fig. 7B).

The basal levels of miR-150-5p in lung tissues and lung endothelium of HFD-fed mice were lower than those of NFD-fed mice (Fig. S4A). After mechanical ventilation, miR-150-5p in lung tissues and lung endothelium of HFD-fed mice were also lower than those of NFD-fed mice. After given miR-150-5p agomir to increase the miR-150-5p in lung tissues and lung endothelium, miR-150-5p agomir also increased VE-cadherin expression in HFD-fed mice by 236%, while this increase was only 60% in NFD-fed mice (Fig. S4B). Multiple immunohistochemistry (IHC) showed that HFD-fed mice had lower expression of VE-cadherin and stronger expression of inflammatory factor TNF-α post mechanical ventilation stimulation and the use of miR-150-5p agomir significantly reversed these changes (Fig. 7C).

VILI induced more severe lung damage in the HFD-fed mice compared to their normal counterparts, characterized by increased vascular permeability, alveolar congestion, alveolar hemorrhage, and inflammatory infiltration. The administration of miR-150-5p agomir substantially ameliorated these pathological changes (Fig. 7D). The lung injury score, W/D weight ratio, BALF cell count, and BALF protein concentration were increased in mechanical ventilation-exposed HFD-fed mice, but these increases were significantly reduced following miR-150-5p agomir treatment (*P* < 0.001, *P* < 0.001, *P* < 0.01, *P* < 0.001, respectively, Fig. 7E–G). Enzyme-linked immunosorbent assay (ELISA) assays of BALF corroborated these findings (Fig. 7H). After miR-150-5p agomir treatment to NFD-fed mice, lung injury score, W/D weight ratio, Evans blue dye leakage, BALF cell count, BALF protein concentration, IL-1β, IL-6, and TNF-α were reduced by 30.6%, 21.5%, 49.5%, 29.0%, 33.1%, 29.1%, 34.1%, and 52.1%, respectively, while after miR-150-5p agomir treatment of HFD-fed mice, these were decreased by 38.5%, 36.9%, 53.5%, 45.8%, 38.5%, 35.8%, 50.6%, and 54.6%, respectively (Fig. S4C–H). These findings suggested that obesity exacerbated the inflammatory response and lung tissue damage, while miR-150-5p agomir administration mitigated VILI.

## Discussion

**Fig. 8:**
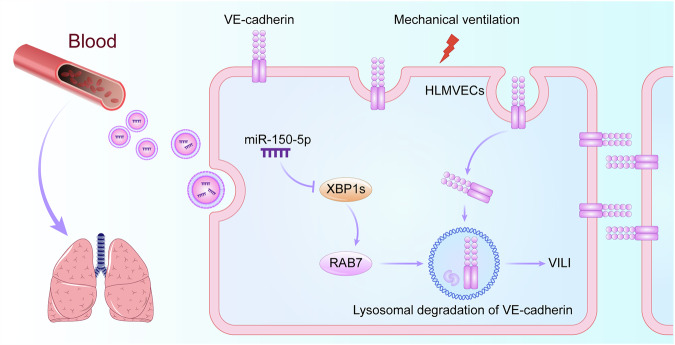
The mechanism by which miR-150-5p in plasma-derived EVs plays a role in obesity aggravating VILI. Obesity leads to a reduction of miR-150-5p in plasma-derived EVs, which is found to target XBP1s and subsequently regulates RAB7, promoting lysosomal degradation of VE-cadherin, leading to increased lung endothelial permeability and aggravating VILI.

In this study, we found that miR-150-5p was decreased in EVs from obese patients and in HLMVECs after cyclic stretch, subsequently upregulating the XBP1s/RAB7 axis and promoting VE-cadherin endocytosis and lysosomal degradation. All these changes ultimately led to further impairment of the endothelial barrier integrity of the lung. Furthermore, our in vivo studies showed that obesity aggravated VILI, but overexpression of miR-150-5p protected pulmonary VE barrier and ameliorated VILI (Fig. 8).

VILI is primarily due to an increased permeability of the pulmonary microvascular endothelium [26]. Ventilation mechanically overstretches alveoli, transmitting pathological stress to the alveolar epithelium and endothelium, compromising the pulmonary endothelial barrier and triggering pulmonary edema [27]. Cell interactions play a crucial role in maintaining endothelial barrier. VE-cadherin is the primary component of endothelial adherent junctions, and its expression is closely linked to intercellular adhesion and the barrier function of endothelial cells [28]. Previous studies indicate that VE-cadherin endocytosis contributes to enhanced endothelial permeability in the LPS-induced ALI model [29, 30], but its specific role in VILI is less understood. Our study demonstrates that cyclic stretch reduced the membrane-bound and total VE-cadherin expression while increased the level of cytoplasmic VE-cadherin, and the cytoplasmic VE-cadherin was degraded through lysosomal pathway.

EVs in patients with lung injury modulated the microvascular endothelial barrier and impacted the prognosis of lung injury [14]. Human umbilical vein endothelial cells display an increased permeability upon exposure to serum derived from obese mice [31]. The direct effects of plasma EVs derived from obese patient on lung endothelial cells during mechanical ventilation have not been previously explored. In this study EVs from obese patients, in particular, enhanced cyclic stretch-induced VE-cadherin endocytosis and degradation, disrupting barrier integrity of HLMVECs. This finding is consistent with previous studies showing decreased barrier function in HLMVECs challenged with plasma EVs in sepsis patients [14].

Our miRNA profile comparing between EVs from normal controls and EVs from obese patients led to a focus on miR-150-5p. In our study, we found that the basal expression level of miR-150-5p in plasma EVs of normal subjects was one of the highest among other miRNAs. This is also supported by another study reported by Chiam et al. [32], which demonstrated that miR-150-5p was one of the top 20 most abundant miRNAs expressed in plasma EVs of healthy individuals. miR-150-5p is an evolutionarily conserved miRNA and its major sources in circulation are T and B lymphocytes [33], whilst it is mainly stored in EVs in plasma [34]. Reduced miR-150-5p in peripheral blood may predict adverse outcomes in a variety of acute and chronic lung diseases [21–23, 35]. The expression levels of miR-150-5p in the LPS-treated HLMVECs were also decreased, and overexpression of miR-150-5p alleviated LPS-induced lung injury in mice [20]. More importantly, various EVs are reported to alleviate sepsis-induced ALI by delivering miR-150-5p [36, 37]. All these previous publications are in line with our data showing the protective effects of miR-150-5p on lungs. Reports showed that XBP1s, a transcription factor exerting its effects through various target genes involving in stress response, has the ability in regulating cell barrier function [24, 38, 39]. miR-150-5p targeted XBP1s, and both miR-150-5p mimics and si-XBP1s reduced VE-cadherin endocytosis and lysosomal degradation, partially restoring endothelial cell barrier function.

The enrichment of DE-miRNAs in regulation of small GTPase mediated signal transduction, regulation of Ras protein signal transduction, and lysosome pathways made us pay attention to the function of RAB7 in HLMVECs. RAB7 is a small GTPase and regulates late endosome trafficking, with significant involvement in autophagosome-lysosome fusion and lysosome maturation. Previous studies demonstrated that elevated RAB7 expression level leads to enhanced lysosomal degradation of mitochondria, low-density lipoprotein, and α-synuclein aggregates [25, 40, 41]. It has been reported that RAB7 facilitates E-cadherin degradation within lysosomes [42]. In this study, RAB7 was identified as a regulator of VE-cadherin lysosomal degradation, supported by our data that RAB7 expression was increased after cyclic stretch or in the cells co-cultured with EVs from obese patients. Besides, RAB7 acts as a downstream of XBP1s, which is a new way we have explored for XBP1s to regulate VE-cadherin [43]. The introduction of miR-150-5p mimics and siRNA targeting XBP1s led to a discernible decrease in RAB7 expression. At the same time, the lysosomal degradation of VE-cadherin was decreased, and the barrier function of endothelial cells was partially restored.

In the VILI mice, we found an increase in the expression of XBP1s and RAB7, which aligned with the findings of our cell experiments. Prolonged mechanical ventilation led to a gradual decrease of VE-cadherin. In addition, we found that obesity aggravated lung endothelial barrier damage in mechanical ventilation mice, manifesting as more severe pulmonary edema, accumulation of inflammatory factors. miR-150-5p agomir treatment attenuated lung injury and pulmonary edema in both control and obese mice, with the therapeutic effect being more pronounced in obese mice, highlighting the therapeutic potential of targeting the XBP1s/RAB7 axis with miR-150-5p.

Despite certain novelties of our work, there are some limitations. We only focused miR-150-5p in our study, and the role of other EVs miRNA on VILI in our experimental setting is unknown and warrants further study. The protective (e.g., preventing leakage) effects of miR-150-5p in cell culture settings were not carried out, but this is well complementally supported by our Evans blue experimental data showing that miR-150-5p protected lung injury and its leakage. In addition, some studies suggested that a HFD-induced obesity can preserve alveolar permeability in VILI models [44], while others showed the increased alveolar neutrophil infiltration in their VILI lung of obese mice [45]. The mechanisms behind these reported previously and our current study are unknown. The difference experimental settings and “disease course” may be the reasons. However, obesity itself increasing pulmonary tissue vascular permeability by down-regulating endothelial adhesion to be critical during the progression of VILI has been well documented previously [31, 46, 47].

In summary, this study sheds light on the EVs-mediated lung injury through which less miR-150-5p modulating the XBP1s/RAB7 pathway in obesity exacerbates VILI. Our work suggests that enhancing miR-150-5p may serve as a novel therapeutic strategy to be developed for VILI prevention and treatment in obese patients.

## Materials and methods

### Human blood samples

After obtaining written informed consent, six obese patients and six healthy volunteers matched with age, gender except normal weight were recruited from The First Affiliated Hospital of Shandong First Medical University. The inclusion criteria for obese subjects included age >18 years old and body mass index (BMI) > 30 kg/m^2^, while the inclusion criteria for control subjects included age >18 years old, BMI < 25 kg/m^2,^ and health. Plasma samples were collected from each subject for extracting EVs for sequencing and subsequent experiments.

### EVs isolation and characterization

EVs were isolated through differential centrifugation. The EVs were analyzed to determine their concentration using the bicinchoninic acid protein analysis kit (Beyotime, Shanghai, China). The expression levels of EVs-positive markers and the EVs-negative marker were assessed through western blot. The morphology of EVs was confirmed using transmission electron microscopy. The particle size distribution and concentration of the EVs were determined through nanoparticle tracking analysis (NTA).

### Cell culture and transfection

HLMVECs were purchased from the American Type Culture Collection (Manassas, VA, USA). HLMVECs were seeded at a density of 5 × 10^5^ cells/mL on collagen I-coated flexible-bottom BioFlex plates (Flexcell, Burlington, NC, USA) and cultured in endothelial cell medium (Sciencell, Carlsbad, CA, USA) supplemented with 1% endothelial cell growth supplement, 1% penicillin/streptomycin solution, and 5% fetal bovine serum (Sciencell, Carlsbad, CA, USA).

HLMVECs were incubated with EVs (20 μg/mL) isolated from normal controls or obese patients for 24 h followed by cyclic stretch in a cyclic manner at a frequency of 30 cycles/min and a 20% range with a stretch-to-relaxation ratio of 1:1 [48]. To explore the VE-cadherin degradation mechanism, MG-132 (10 µM; EMD Milliporeand, Shanghai, China), chloroquine (10 µM; Sigma-Aldrich, St. Louis, MO, USA), and bafilomycin A1 (100 nM; Sigma-Aldrich, St. Louis, MO, USA) were used for pretreatment for 2 h [49]. siRNA of XBP1s and siRNA naive control (si-NC), RAB7 plasmid and plasmid NC, miR-150-5p mimics and mimics NC were transfected into recipient cells using lipofectamine 3000 (Invitrogen, Carlsbad, CA, USA) as per the manufacturer’s instructions. The sequences of miR-150-5p mimics and XBP1s siRNA are as follows:

miR-150-5p mimics’ sequence: 5′-UCUCCCAACCCUUGUACCAGUG-3′

XBP1s siRNA #1 forward: 5′-GGAACAGCAAGUGGUAGAUTT-3′

XBP1s siRNA #1 reverse: 5′-AUCUACCACUUGCUGUUCCTT-3′

XBP1s siRNA #2 forward: 5′-GGAGCUGGGUAUCUCAAAUTT-3′

XBP1s siRNA #2 reverse: 5′-AUUUGAGAUACCCAGCUCCTT-3′

XBP1s siRNA #3 forward: 5′-GCUUGGUGUAAACCAUUCUTT-3′

XBP1s siRNA #3 reverse: 5′-AGAAUGGUUUACACCAAGCTT-3′

### EVs uptake assay

EVs (200 μL) were resuspended in 200 μL diluteC and PKH67 (Sigma-Aldrich, St Louis, MO) was diluted with diluteC at a ratio of 100:1–200 μL. The above two were mixed and allowed to stand for 5 min. An equal volume of 5% bovine serum albumin (BSA) was added to the mixture and then centrifuged at 120,000 × *g*, 4 °C for 70 min. After discarded supernatant, EVs were resuspended in PBS and then added to the cell culture medium. After HLMVECs were co-cultured with the mixture for 4 h, 4′,6-Diamidino-2-phenylindole (DAPI) and fibrin actin (F-actin) were used to stain nucleus and cytoplasm, respectively. Micrographs were taken under a fluorescence microscope.

### EVs miRNA sequencing

Plasma samples from normal controls and obese patients were sent to Genesky Biotechnology Co. Ltd (Shanghai, China) for miRNA sequencing. Total RNA of EVs was extracted from plasma samples, and its concentration was determined before subjected to next-generation sequencing using the Illumina MiSeq platform. DE-miRNAs were identified with thresholds set at an absolute log2Fold (FC) > 1, and a *p*-value < 0.05 after data preprocessing.

### Immunofluorescent staining

After cyclic stretch challenge, HLMVECs were fixed with 4% paraformaldehyde for 20 min, permeabilized with Saponin (Beyotime, Shanghai, China) for 10 min, and blocked in 5% BSA for 30 min. Subsequently, the cells were incubated overnight at 4 °C with primary antibodies and incubated with red or green fluorescent secondary antibodies for 1 h at room temperature. Finally, the nuclei were stained with DAPI for 5 min and then assessed under confocal microscope (Nikon, Tokyo, Japan). All antibodies used in this study are listed in Supplementary Table S1.

### LDH release

LDH release indicating reputed cell membrane was determined using a LDH Assay Kit (Beyotime, Shanghai, China) according to the manufacturer’s instructions.

### Luciferase assay

To explore the targeting of XBP1s by miR-150-5p, HEK 293 T cells were co-transfected with luciferase vectors containing wild-type (WT) or mutant (MUT) 3ʹ-UTR of XBP1s and miR-150-5p mimics or NC mimics using lipofectamine 3000 reagent (Invitrogen,Carlsbad, CA, USA). To explore the transcriptional regulation of RAB7 by XBP1s, a 726 bp fragment containing RAB7 promoter to the XBP1s binding site was cloned into the pGL3-basic luciferase reporter vector WT RAB7, and a binding site mutation vector MUT RAB7 was created. WT or MUT RAB7 luciferase reporter and XBP1s overexpression or control plasmids were co-transfected into HEK 293T cells using Lipofectamine 3000 reagent. After 48 h of transfection, luciferase activity was measured with the dual luciferase reporter gene detection system (Beyotime, Shanghai, China).

### Co-IP

Following the cyclic stretch treatment, the cells were lysed in IP-buffer (87787, Thermo Fisher Scientific, Waltham, MA, USA), and subsequently the lysate was centrifuged at 12,000 × *g* rpm at 4 °C for 15 min. A small portion of the supernatant was reserved for use as input, while the remaining supernatant was combined with protein A/G Beads for immunoprecipitation, along with the corresponding antibodies and homologous IgG. This mixture was then incubated overnight at 4 °C with gentle rotation. The beads were washed five times with IP-buffer, and the immunoprecipitated proteins were reconstituted in 2× protein loading buffer for western blot analysis.

### Animal model

C57BL/6 WT mice (5–6 weeks old, weighing 16–20 g) were obtained from Vital River Laboratory (Beijing, China) and maintained in a pathogen-free environment with stable temperature and humidity (22 ± 2 °C, 50 ± 10%) and 12-h light/dark cycle and had a free access to water and food. Mice were randomly assigned to groups without blinding of group assignment. For the obese mouse model, a HFD (Research Diets, D12492, 60% cal% fat) was fed for 9 weeks, and the control mice were fed a NFD (Research Diets, D12450B, 10 cal% fat).

Animal models were established based on previous studies [50]. Mice were anesthetized with an intraperitoneal injection of pentobarbital sodium (60 mg/kg) and received mechanical ventilation treatment for 2 or 4 h via tracheotomy and intubation. The ventilator parameters were as follows: tidal volume 28 mL/kg, respiratory rate 60 breaths/min, end-expiratory pressure 0 cm H_2_O. After mechanical ventilation, the mice were euthanized with an intraperitoneal injection of an overdose of anesthetic, and lung tissues and BALF were collected for subsequent experiments.

For in vivo experiments with EVs injection, 100 µg of EVs from different sources were injected into each mouse through the tail vein, followed by mechanical ventilation treatment 24 h later. To verify the role of miR-150-5p, before mechanical ventilation treatment, miR-150-5p agomir and agomir NC (5 nmol dissolved in 40 μL saline; RiboBio, Guangzhou, China) were intranasally administered to mice. To obtain mice with low expression of miR-150-5p, 150-5p antagomir and antagomir NC (3 nmol dissolved in 100 μL saline; RiboBio, Guangzhou, China) was injected into the tail vein twice a week for 4 weeks.

### Isolation of pulmonary endothelial cells

Lung tissues obtained from treated mice in each group were minced and digested for 90 min and then filtered through a 70 µm cell strainer. Lung endothelial cells were sorted twice using CD45 and CD31 microbeads (Miltenyi Biotec Inc., Auburn, CA, USA) as described previously [46].

### Hematoxylin-eosin (HE) staining and lung injury scores

To assess the morphological changes in lung tissues, the lung specimens were fixed in 4% formalin, embedded in paraffin, and subsequently cut into 5 μm sections. These sections were then stained with HE and examined under a light microscope (Nikon, Tokyo, Japan). To determine the lung injury in mice, a scale ranging from 0 to 4 was employed to classify the severity of lung injury [51].

### W/D weight ratio

The severity of pulmonary edema was assessed using the W/D weight ratio. The right lung tissue of the mouse was extracted, and any surface moisture was carefully removed with gauze. Subsequently, the wet weight of the lung was measured. Next, the lung tissue was dried in an oven at 60 °C for 48 h and weighed again to determine the dry weight. Finally, the W/D weight ratio was calculated.

### Evans blue staining

Mice were injected at the end of the experiments with 20 mg/kg Evans blue dye (Sigma-Aldrich, St. Louis, MO, USA) through the tail vein. They were euthanized 1 h later, lung tissues were removed and weighed, and then homogenized and incubated with formamide at 60 °C for 12 h. After centrifugation, the optical density of the supernatant was measured with a microplate reader (absorbance at 620 nm). Results are expressed as micrograms of dye per gram of tissue.

### BALF cell count and total protein quantification

The BALF samples were centrifuged at 500 × *g* for 5 min at 4 °C, the supernatant was collected, and the protein level was measured by bicinchoninic acid protein analysis kit. The cell pellet was resuspended and the total cell number was determined with a cell counting chamber under a microscope.

### ELISA

The levels of IL-1β, IL-6, and TNF-α in the BALF were then assessed using ELISA kits (ReedBiotech, Wuhan, China) following the instructions provided by the manufacturer.

### Multiplex IHC

Lung tissues were sectioned at thickness of 4 μm. Deparaffinization of tissue sections was done through xylenes and rehydration through decreasing graded alcohol. AR6 buffer (Akoya Biosciences, Marlborough, MA, USA) was used for antigen retrieval. Endogenous peroxidase was inactivated by incubation in 3% H_2_O_2_ for 10 min. Multiplex IHC was performed by several rounds of staining, each including a protein block with 1% BSA followed by primary antibody and corresponding secondary horseradish peroxidase-conjugated antibody against mouse or rabbit immunoglobulins (Supplementary Table S1). The slides were then incubated in different Opal fluorophore (1:100) diluted in 1 × plus amplification diluent. After tyramide signal amplification and covalent linkage of the individual opal fluorophores to the relevant epitope or epitopes, the primary and secondary antibodies were removed via antigen retrieval as previously mentioned, and the next cycle of immunostaining was initiated. All slides were counterstained with DAPI and mounted with anti-fade fluorescence mounting medium (ab104135, Abcam, Cambridge, MA, USA).

### Western blot

After in vivo and in vitro experimental treatments, proteins were extracted from lung tissues and cells. Membrane and cytoplasmic proteins were obtained through the use of a membrane and cytoplasmic protein extraction kit (Beyotime, Shanghai, China). The protein concentration was determined by employing the bicinchoninic acid protein analysis kit. Equal amounts of proteins were separated using 10% SDS polyacrylamide gels and subsequently transferred to polyvinylidene difluoride membranes. Following this, the membranes were blocked with 5% BSA and then incubated with the primary antibody overnight at 4 °C. Then the membranes were incubated with secondary antibody. A protein imaging system (Bio-Rad, Hercules, CA, USA) was used to detect protein imaging, and the quantitative analysis was performed using ImageJ software (National Institutes of Health, NIH, USA). All antibodies used are listed in Supplementary Table S1.

### RT-qPCR

Total RNA was extracted from lung tissues or cells using the Fastagen RNA extraction kit (Shanghai, China), and cDNA was synthesized with PrimeScript™ RT reagent Kit (TaKaRa, Tokyo, Japan) following the manufacturer’s instructions. RT-qPCR analysis was conducted on a Light Cycler instrument (Bio-Rad, California, USA) with the FastStart Essential DNA Green Master Kit (Roche, Basel, Switzerland). The expression data were normalized to the mRNA level of GAPDH or the miRNA U6. Each sample was loaded in triplicate and analyzed using the 2^−ΔΔCt^ method. The sequences of the primers used are as follows:

VE-cadherin forward: 5′-GCATCGGTTGTTCAATGCGT-3′

VE-cadherin reverse: 5′-CGCTTCCACCACGATCTCAT-3′

XBP1s forward: 5′-GTCCGCAGCACTCAGACTAC-3′

XBP1s reverse: 5′-CTCTGGGGAAGGGCATTTGA-3′

RAB7 forward: 5′-GTTCCAGTCTCTCGGTGTGG-3′

RAB7 reverse: 5′-TTGAATGTGTTGGGGGCAGT-3′

GAPDH forward: 5′-GCACCGTCAAGGCTGAGAAC-3′

GAPDH reverse: 5′-TGGTGAAGACGCCAGTGGA-3′

Has-miR-150-5p forward: 5′-CAGTATTCTCTCCCAACCCTTGTA-3′

Has-miR-150-5p reverse: 5′-TATGGTTTTGACGACTGTGTGAT-3′

U6 forward: 5′-CAGCACATATACTAAAATTGGAACG-3′

U6 reverse: 5′-ACGAATTTGCGTGTCATCC-3′

### Statistical analysis

Data were presented as mean ± standard deviation (SD). All experiments in this study were repeated independently at least 3 times. Student’s *t* test or one-way analysis of variance (ANOVA) followed by Tukey post hoc test was conducted utilizing Prism 8.0 software (GraphPad Software, Durham, NC, USA). A statistically significant difference was considered when a value P is less than 0.05.

## Supplementary information

Supplementary Table S1

Supplementary Figure caption

Supplementary Figure S1

Supplementary Figure S2

Supplementary Figure S3

Supplementary Figure S4

Full length western blots

## Data Availability

The datasets used or/and analyzed during the current study are available from the corresponding author on reasonable request.
