# Supplementary material for: Obesity-associated reduction of miR-150-5p in extracellular vesicles promotes ventilator-induced lung injury by modulating the lysosomal degradation of VE-cadherin
Source: Cell Death Discov. 2025 May 6;11:220. doi: 10.1038/s41420-025-02499-5 (PMC12055972; doi:10.1038/s41420-025-02499-5)
Supplement: Supplementary file 1 — Supplementary Table S1 [file 41420_2025_2499_MOESM1_ESM.docx]

**Supplementary Table S1. Antibodies used in this study**

| Antibody name | Company | Catalog number | Source | Reactive | Dilution |
| --- | --- | --- | --- | --- | --- |
| CD63 | Santa Cruz | sc-5275 | Mouse | M, H, R | WB 1:200 |
| Alix | Santa Cruz | sc-53540 | Mouse | M, H, R | WB 1:200 |
| Calnexin | Santa Cruz | sc-23954 | Mouse | M, H, R | WB 1:200 |
| Apolipoprotein A1 | Santa Cruz | Sc-376818 | Mouse | M, H, R | WB 1:200 |
| GAPDH | CST | #2118 | Rabbit | M, H, R | WB 1:1000 |
| Na,K-ATPase | CST | #3010 | Rabbit | M, H, R | WB 1:1000 |
| VE-cadherin | CST | #2500 | Rabbit | H | WB 1:1000 |
| VE-cadherin | Santa Cruz | sc-9989 | Mouse | M, H, R | WB 1:200 |
| XBP1s | CST | #12782 | Rabbit | H | WB 1:1000 |
| XBP1s | Proteintech | 24868-1-AP | Rabbit | M, H, R | WB 1:1000 |
| RAB7 | Abcam | ab137029 | Rabbit | M, H | WB 1:1000 |
| ZO1 | Proteintech | 21773-1-AP | Rabbit | M, H, R | WB 1:5000 |
| Occludin | Proteintech | 66378-1-Ig | Mouse | M, H | WB 1:5000 |
| VE-cadherin | CST | #2500 | Rabbit | H | IF 1:400 |
| RAB7 | Santa Cruz | sc-376362 | Mouse | M, H, R | IF 1:50 |
| LAMP1 | Santa Cruz | sc-20011 | Mouse | M, H, R | IF 1:50 |
| CD31 | Abcam | ab182981 | Rabbit | M, H, R | Multiplex IHC1:2000 |
| VE-cadherin | Santa Cruz | sc-9989 | Mouse | M, H, R | Multiplex IHC 1:50 |
| LAMP1 | Santa Cruz | sc-20011 | Mouse | M, H, R | Multiplex IHC 1:50 |
| TNF-α | Abcam | ab34674 | Rabbit | M | Multiplex IHC 1:200 |
