# Supplementary material for: Obesity-associated reduction of miR-150-5p in extracellular vesicles promotes ventilator-induced lung injury by modulating the lysosomal degradation of VE-cadherin
Source: Cell Death Discov. 2025 May 6;11:220. doi: 10.1038/s41420-025-02499-5 (PMC12055972; doi:10.1038/s41420-025-02499-5)
Supplement: Supplementary file 2 — Supplementary Figure caption [file 41420_2025_2499_MOESM2_ESM.docx]

**Supplementary Figures**


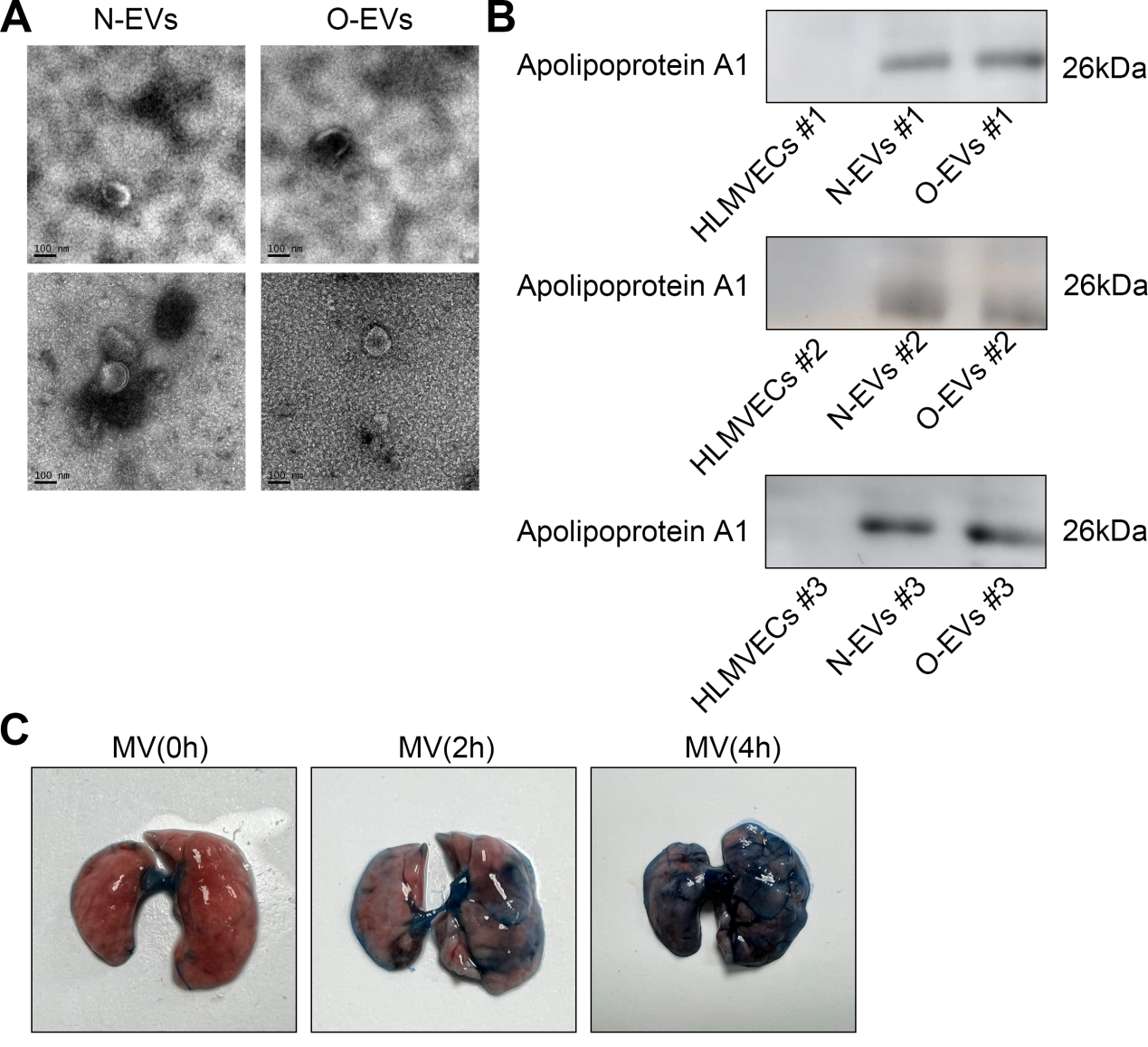


**Fig. S1. TEM images and detection of APOA1 in EVs from normal controls and EVs from obese patients.**

**(A)** TEM images of EVs from normal controls and EVs from obese patients.

**(B)** Apolipoprotein A1 in EVs from normal controls and EVs from obese patients detected via western blot.

**(C)** Representative images of Evans blue dye leakage in the lung.

MV, mechanical ventilation; N-EVs, EVs from normal controls; O-EVs, EVs from obese patients.


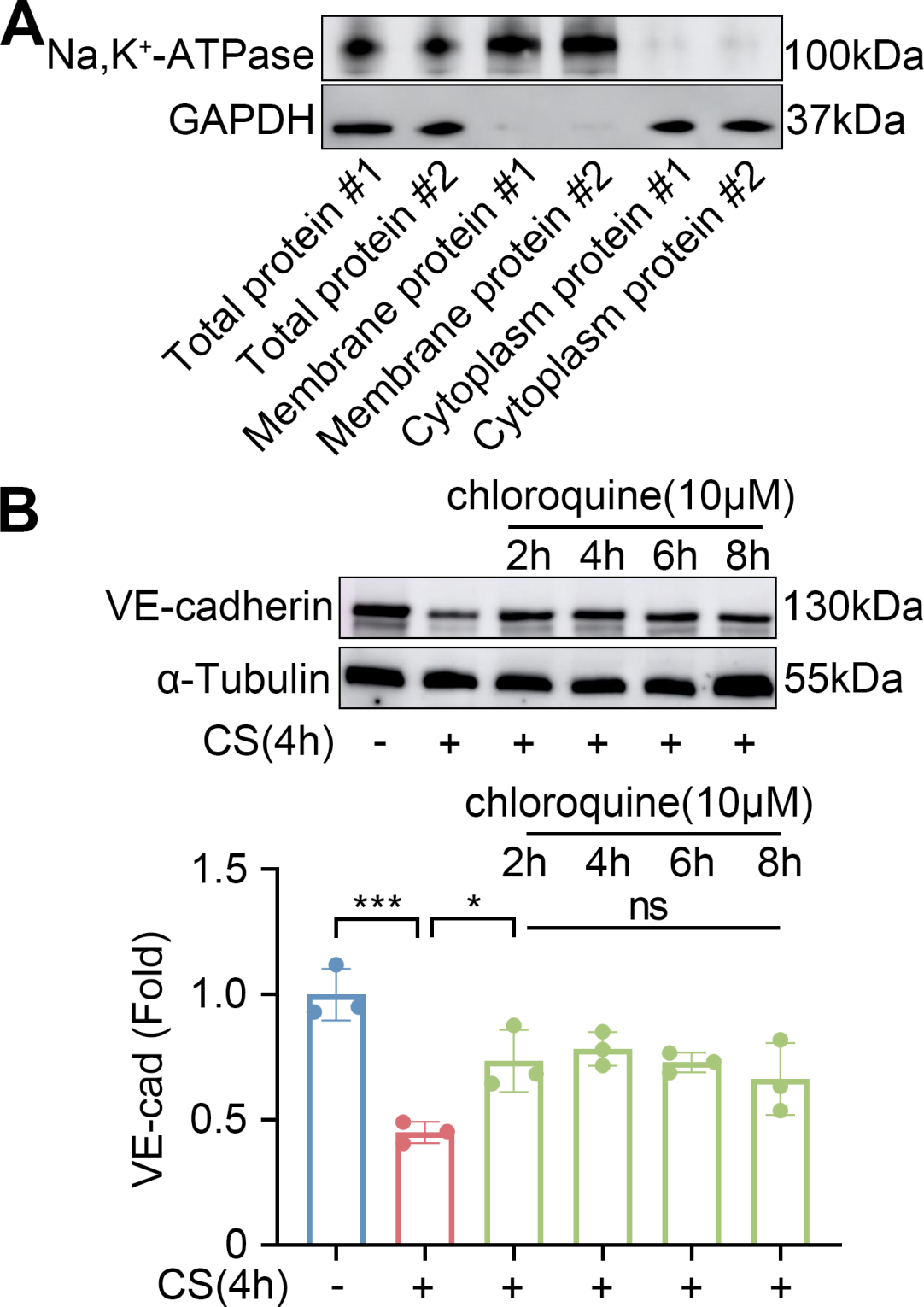


**Fig. S2. Validation of membrane protein and plasma protein extraction and chloroquine pretreatment.**

**(A)** Validation of membrane protein and plasma protein extraction via western blot.

**(B)** Validation of chloroquine pretreatment (n=3).

Data are presented as the mean ± SD; *P < 0.05, ** P < 0.01, *** P < 0.001, ns: not significant; CS, cyclic stretch; N-EVs, EVs from normal controls; O-EVs, EVs from obese patients.


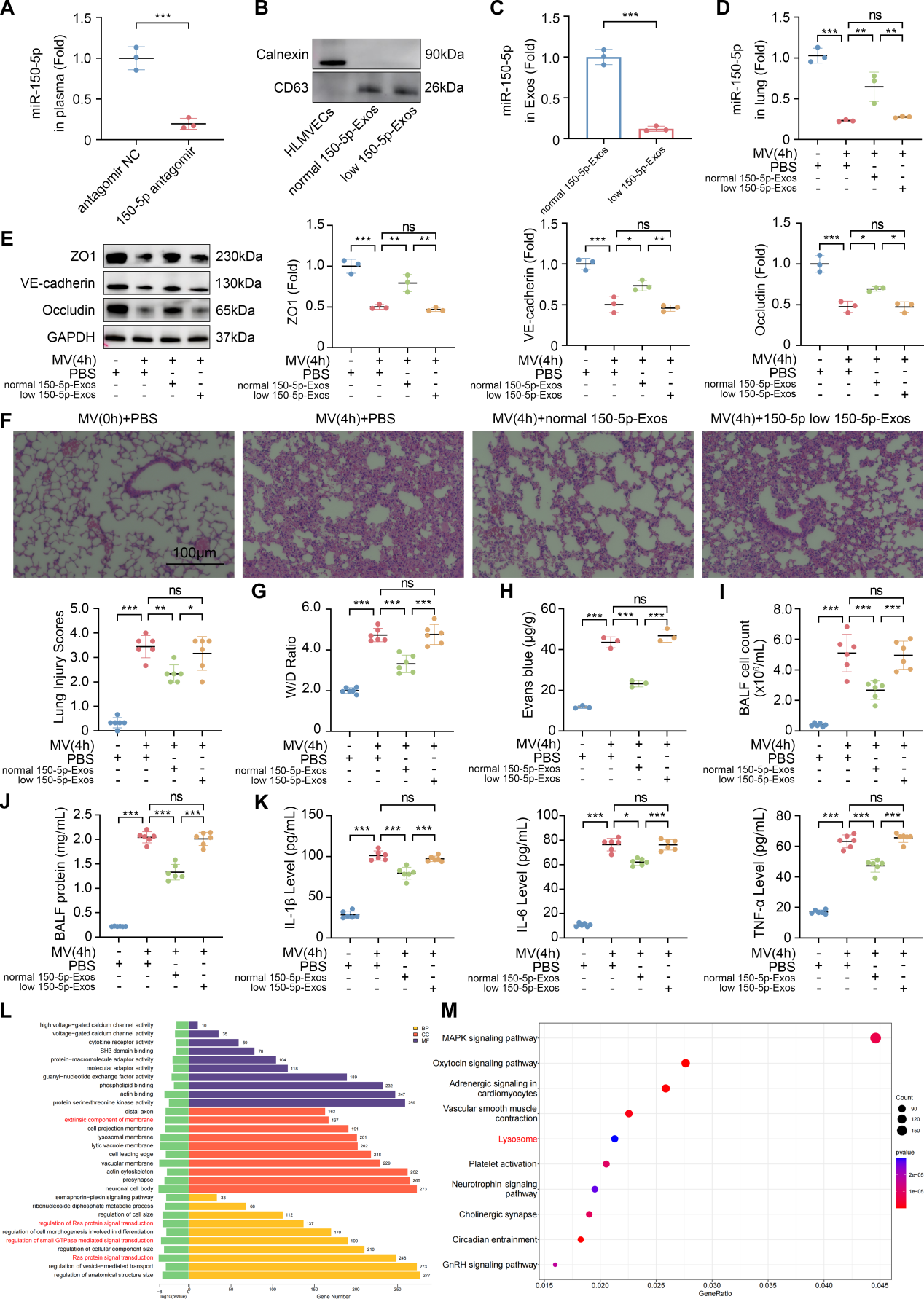


**Fig. S3. Lung protective effect of miR-150-5p in circulating EVs.**

**(A)** Quantitative analysis of miR-150-5p expression levels in plasma of mice injected with antagomir NC and mice injected with miR-150-5p antagomir (n=3).

**(B)** EVs representative markers detected via western blot.

**(C)** miR-150-5p expression levels in EVs from mice injected with antagomir NC and mice injected with miR-150-5p antagomir (n=3).

**(D)** Quantitative analysis of miR-150-5p expression levels in lung tissues of different groups of mice (n=3).

**(E)** Western blot analysis of VE-cadherin, ZO1 and Occludin expression levels in lung tissues of different groups of mice (n=3).

**(F)** Lung injury scores of mice of different groups (n=6).

**(G)** Lung W/D ratio of mice of different groups (n=6).

**(H)** Lung Evans blue dye leakage of mice of different groups (n=3).

**(I)** BALF cell count of mice of different groups (n=6).

**(J)** BALF protein concentration of mice of different groups (n=6).

**(K)** The levels of IL-1β, IL-6, TNF-α in BALF of mice of different groups (n=6).

**(L)** GO enrichment analysis based on DE-miRNAs.

**(M)** KEGG enrichment analysis based on DE-miRNAs.

Data are expressed as the mean ± SD; *P < 0.05, ** P < 0.01, *** P < 0.001, ns: not significant; MV, mechanical ventilation; normal 150-5p-Exos, EVs from mice injected with antagomir NC; low 150-5p-Exos, EVs from mice injected with miR-150-5p antagomir; BP, biological process; MF, molecular function; CC, cellular component.


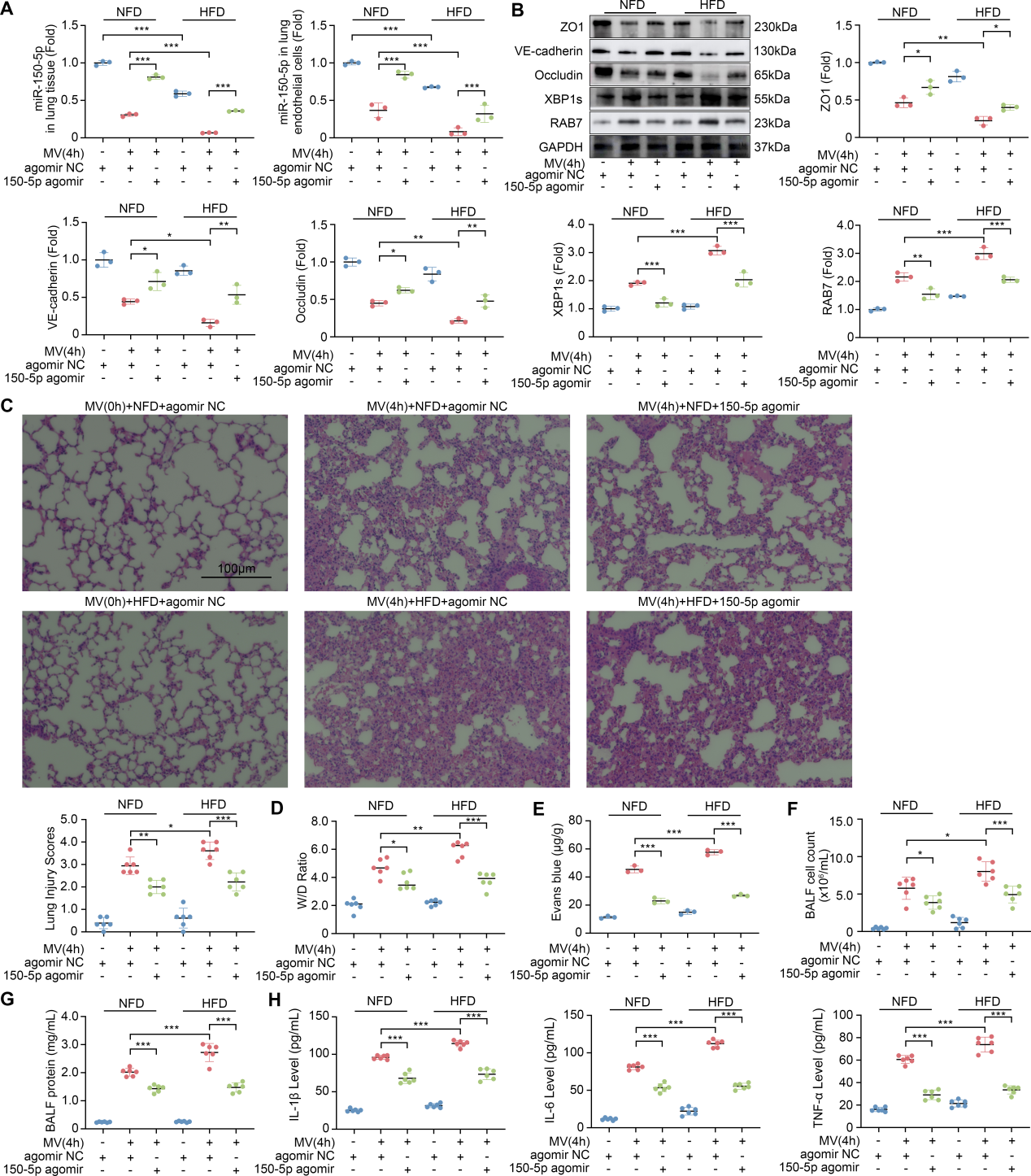


**Fig. S4. The therapeutic effect of miR-150-5p agomir on NFD-fed mice and HFD-fed mice.**

**(A)** Quantitative analysis of miR-150-5p expression levels in lung tissues in lung tissues and lung endothelium of NFD-fed mice and HFD-fed mice subjected to 4-h mechanical ventilation (n=3).

**(B)** Western blot analysis of VE-cadherin, ZO1, Occludin, XBP1s and RAB7 in NFD-fed mice and HFD-fed mice subjected to 4-h mechanical ventilation (n=3).

**(C)** Lung injury scores of NFD-fed mice and HFD-fed mice subjected to 4-h mechanical ventilation (n=6).

**(D)** Lung W/D ratio of NFD-fed mice and HFD-fed mice subjected to 4-h mechanical ventilation (n=6).

**(E)** Lung Evans blue dye leakage of NFD-fed mice and HFD-fed mice subjected to 4-h mechanical ventilation (n=3).

**(F)** BALF cell count of NFD-fed mice and HFD-fed mice subjected to 4-h mechanical ventilation (n=6).

**(G)** BALF protein concentration of NFD-fed mice and HFD-fed mice subjected to 4-h mechanical ventilation (n=6).

**(H)** The levels of IL-1β, IL-6, TNF-α in BALF of NFD-fed mice and HFD-fed mice subjected to 4-h mechanical ventilation (n=6).

Data are expressed as the mean ± SD; *P < 0.05, ** P < 0.01, *** P < 0.001, ns: not significant; MV, mechanical ventilation; NFD,normal fat diet; HFD, high fat diet.
