# Supplementary figures and images for: Obesity-associated reduction of miR-150-5p in extracellular vesicles promotes ventilator-induced lung injury by modulating the lysosomal degradation of VE-cadherin
Source: Cell Death Discov. 2025 May 6;11:220. doi: 10.1038/s41420-025-02499-5 (PMC12055972; doi:10.1038/s41420-025-02499-5)

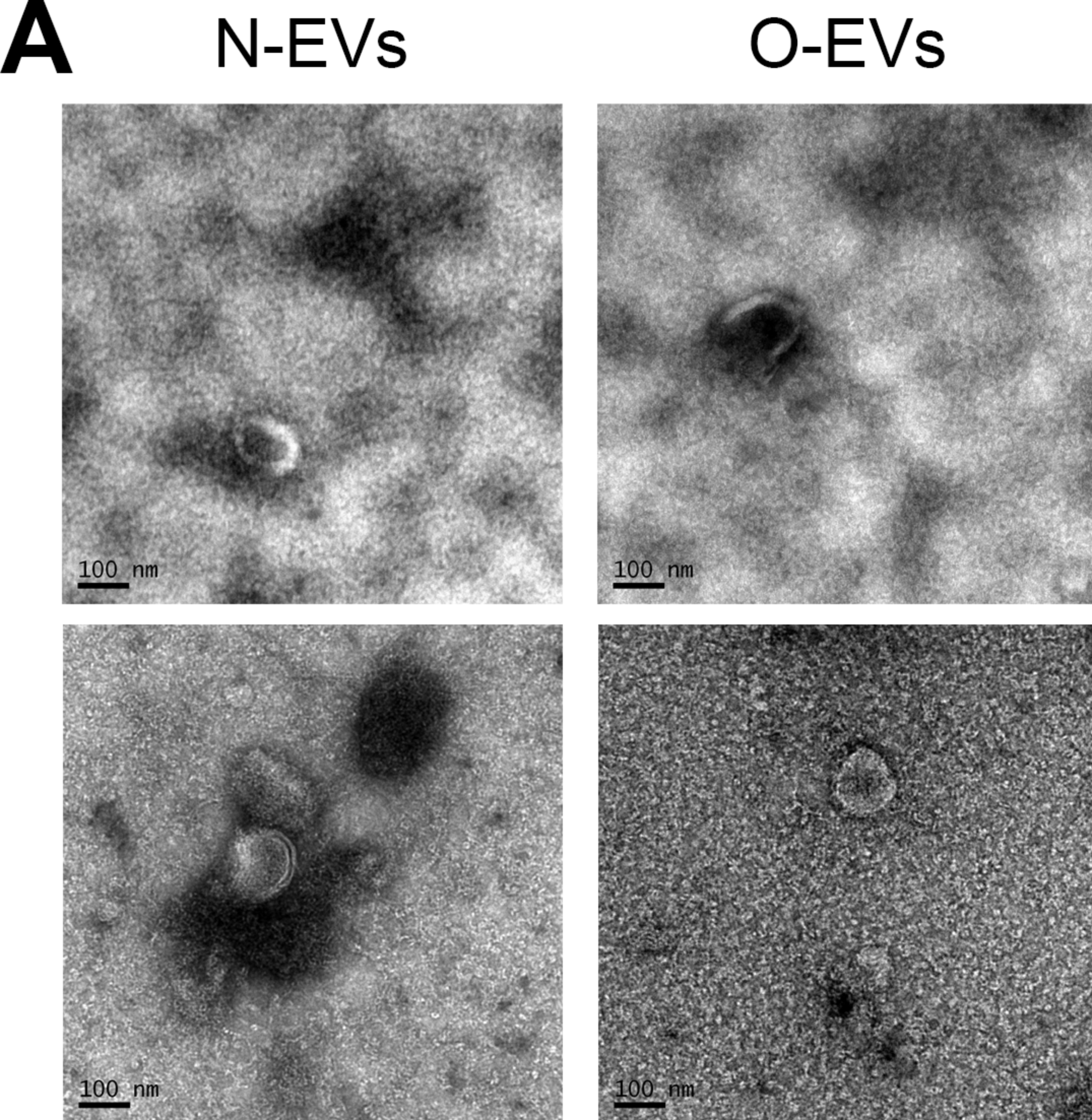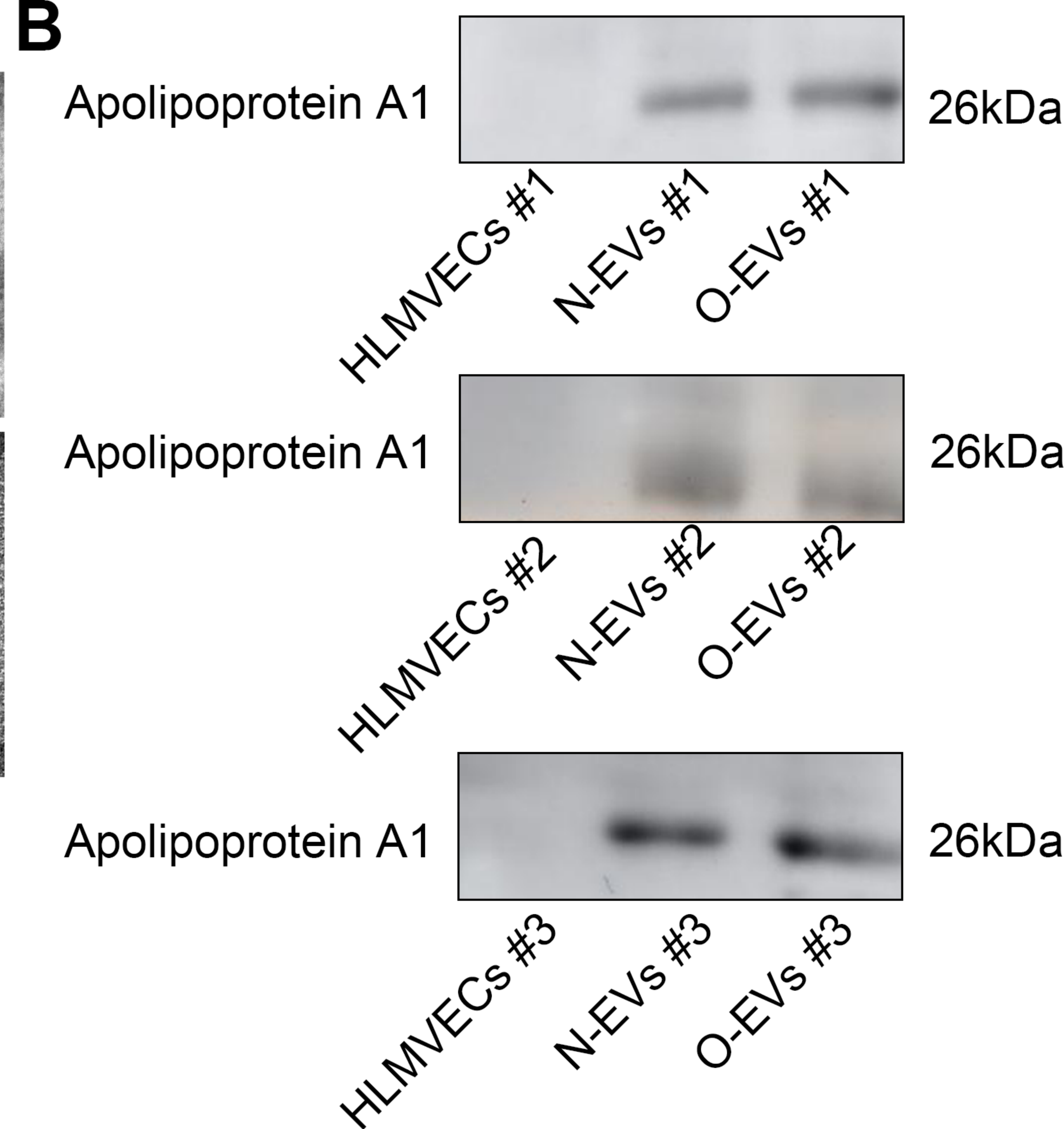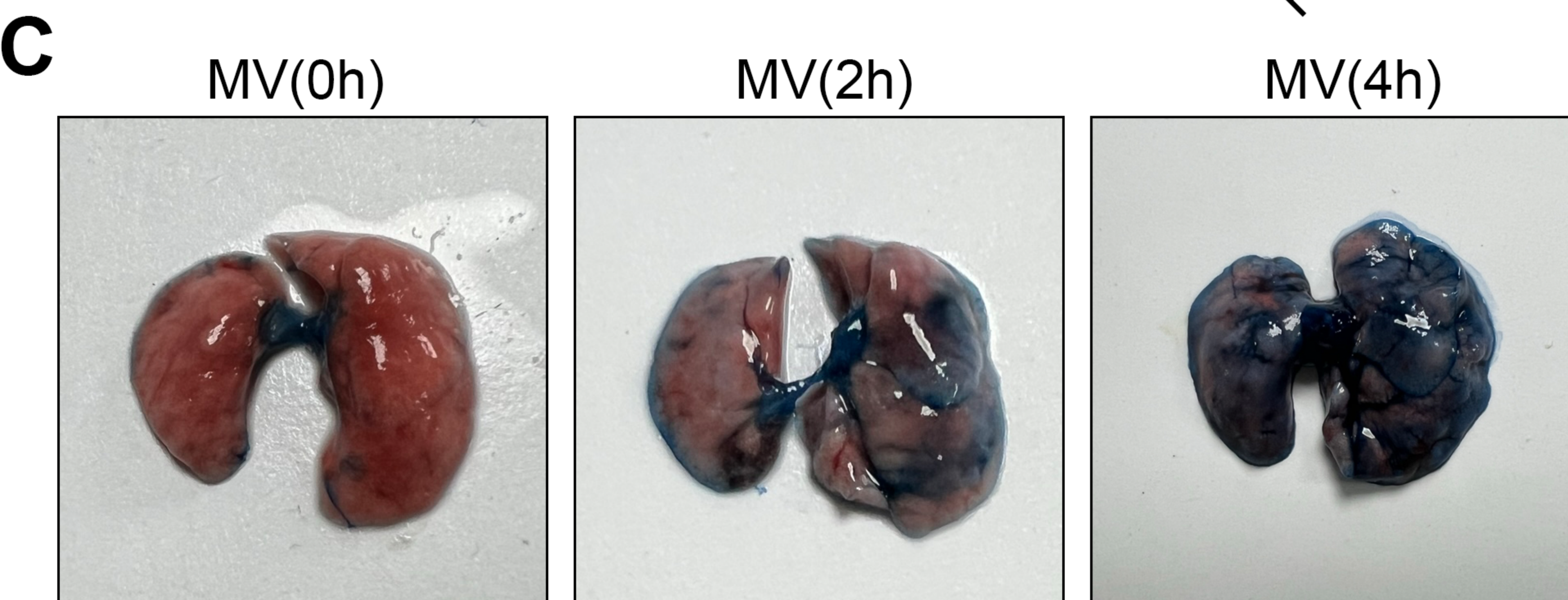

Supplement: Supplementary file 3 — Supplementary Figure S1 [file 41420_2025_2499_MOESM3_ESM.pdf]

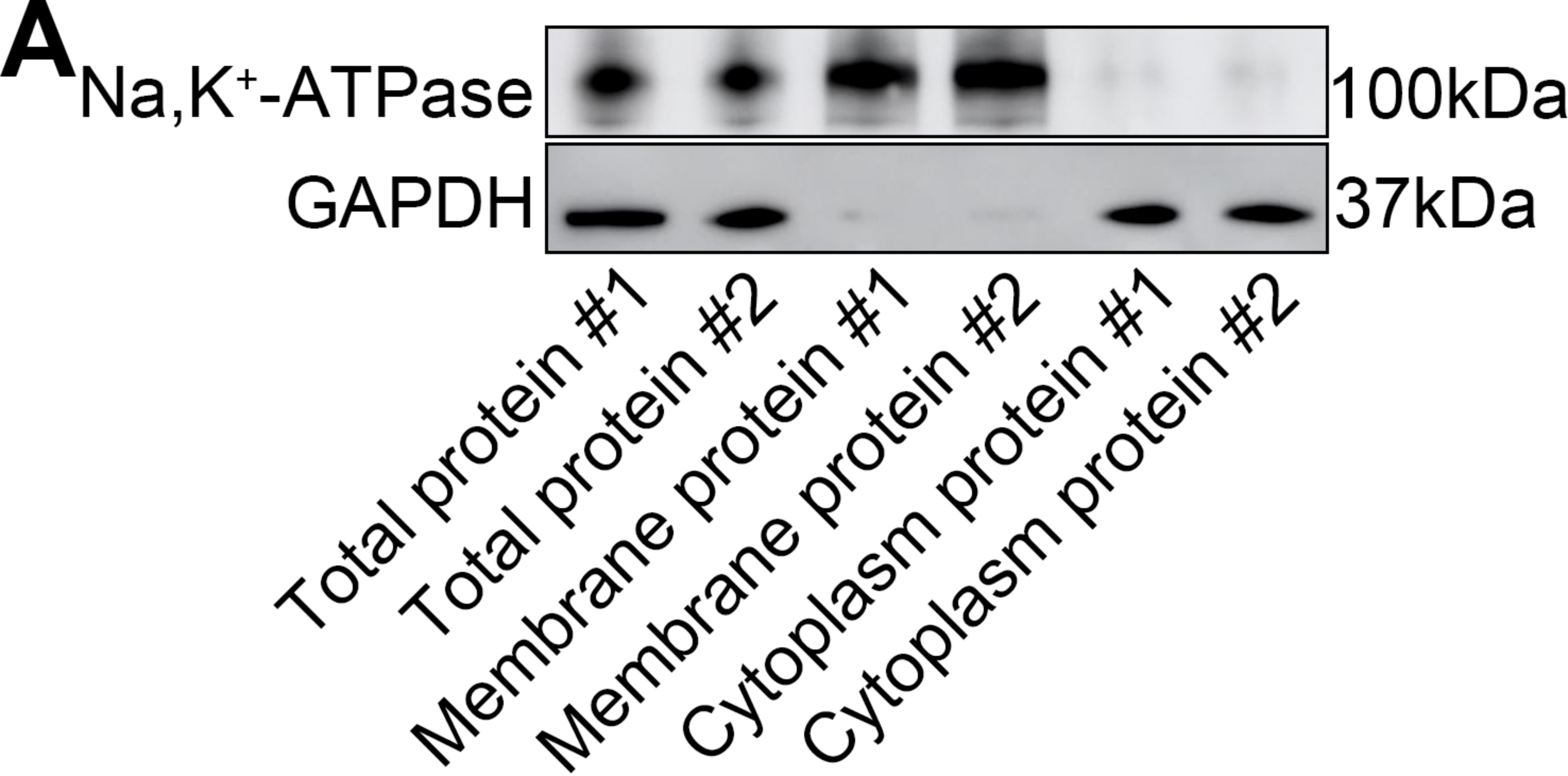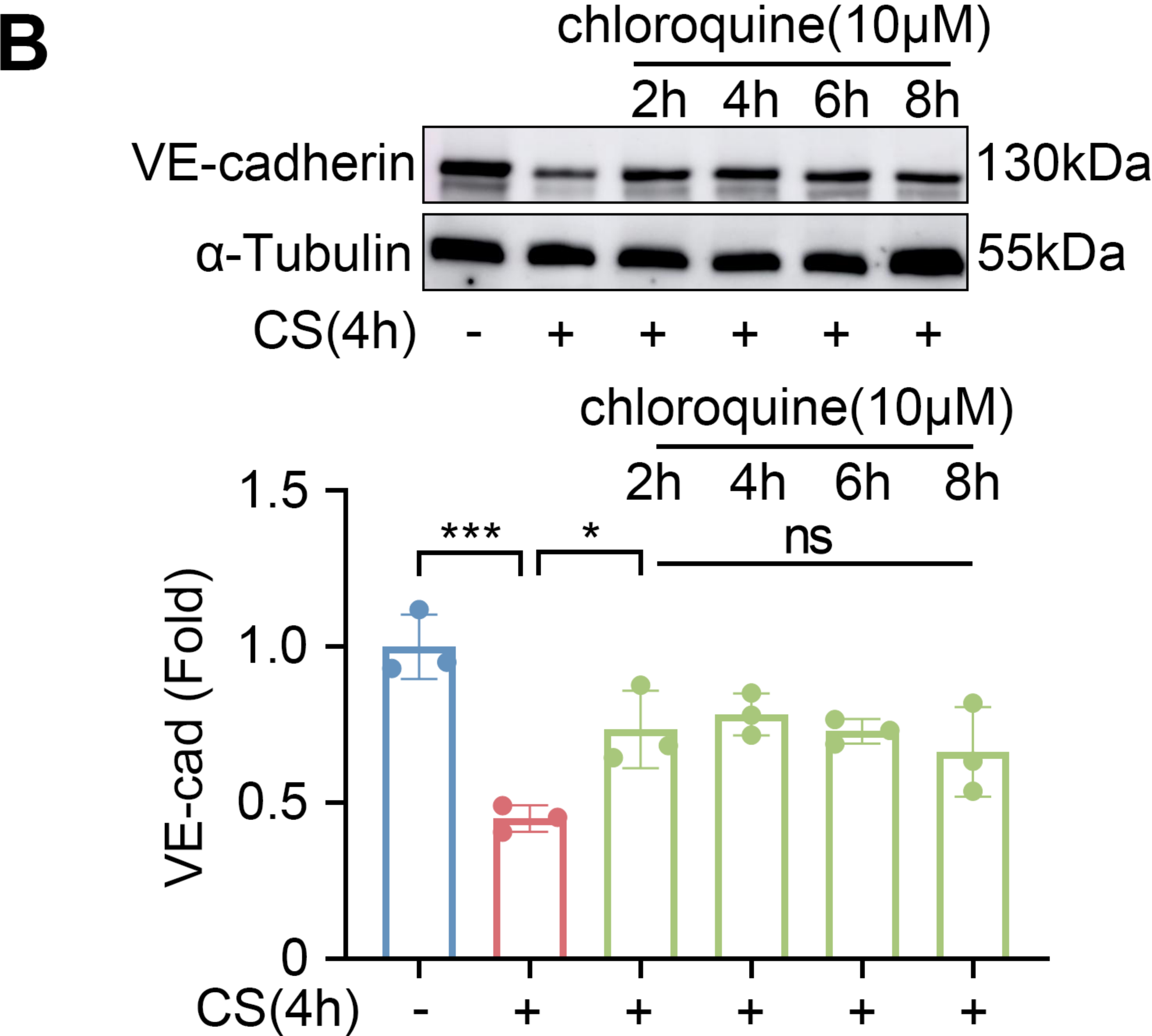

Supplement: Supplementary file 4 — Supplementary Figure S2 [file 41420_2025_2499_MOESM4_ESM.pdf]

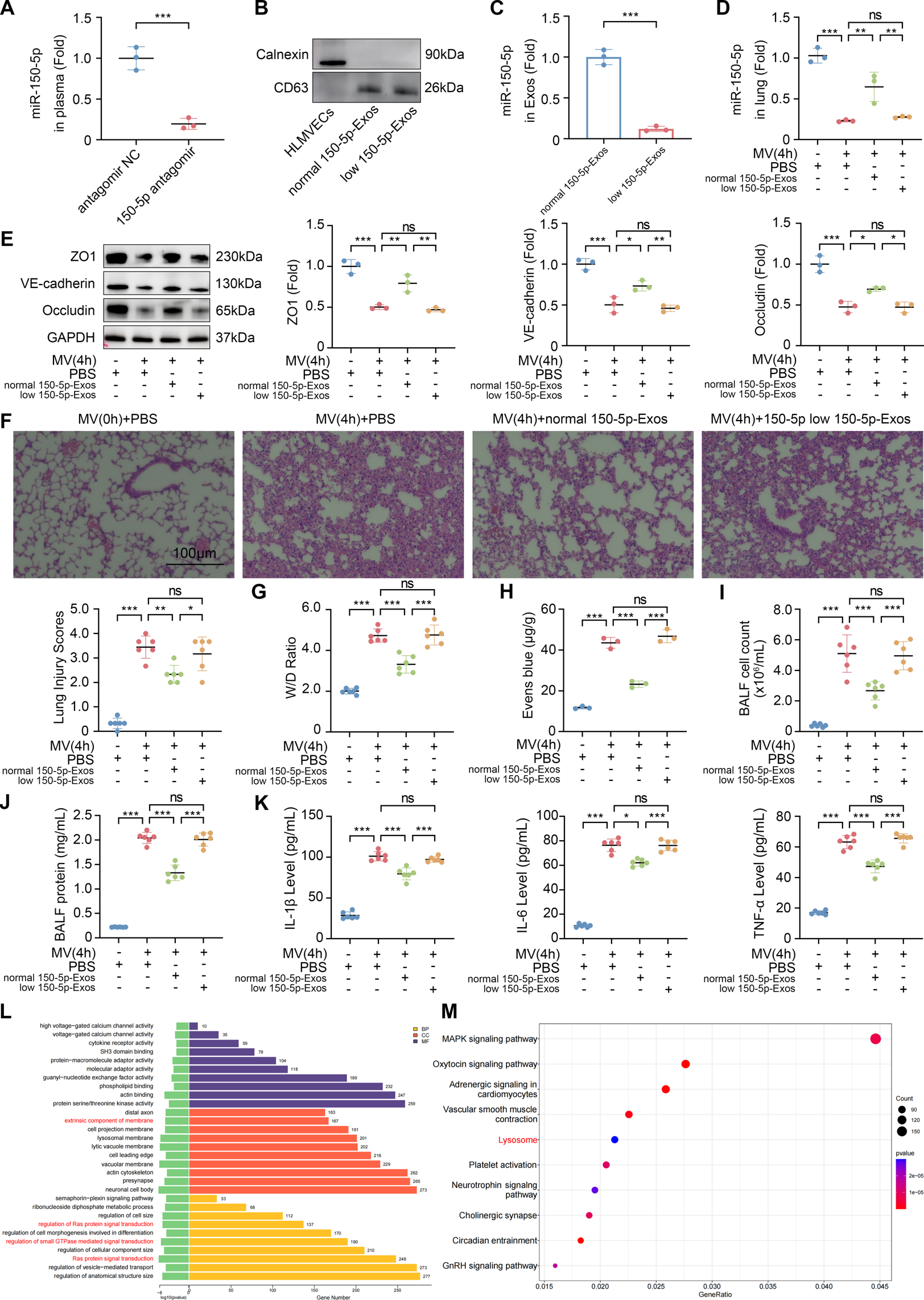

Supplement: Supplementary file 5 — Supplementary Figure S3 [file 41420_2025_2499_MOESM5_ESM.pdf]

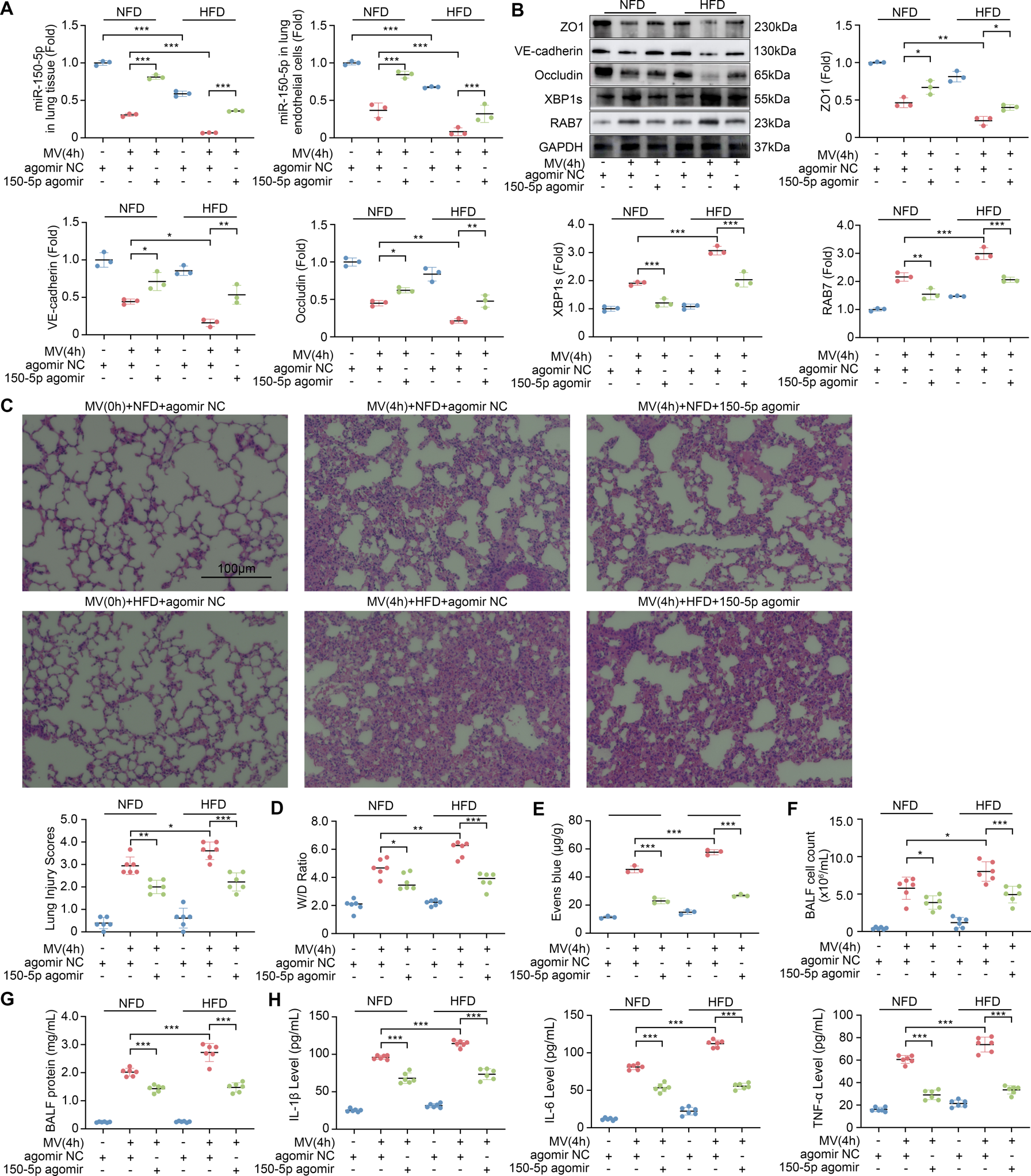

Supplement: Supplementary file 6 — Supplementary Figure S4 [file 41420_2025_2499_MOESM6_ESM.pdf]
