# Supplementary material for: Obesity-associated reduction of miR-150-5p in extracellular vesicles promotes ventilator-induced lung injury by modulating the lysosomal degradation of VE-cadherin
Source: Cell Death Discov. 2025 May 6;11:220. doi: 10.1038/s41420-025-02499-5 (PMC12055972; doi:10.1038/s41420-025-02499-5)

Figure1.E

EVs markers-CD63 Repeat1

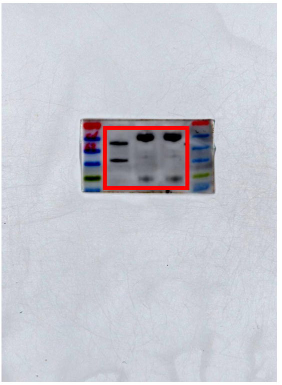

Figure1.E

EVs markers-Calnexin

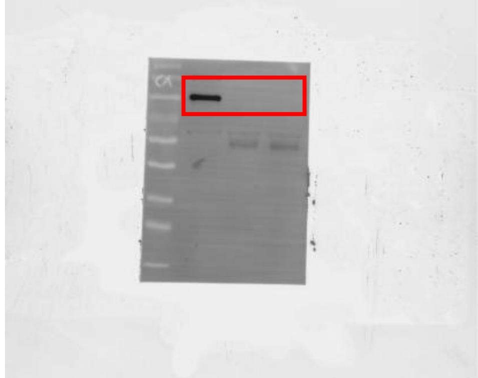

Figure1.E

EVs markers-Alix

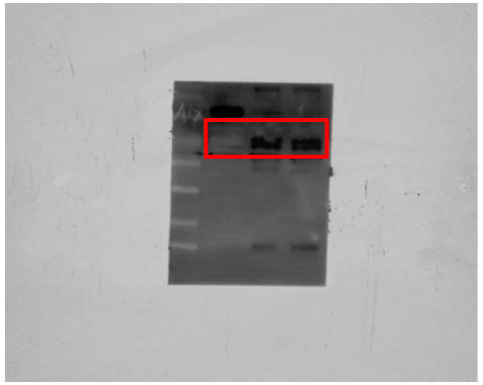

Figure2.A
Total VE-cadherin and
corresponding GAPDH

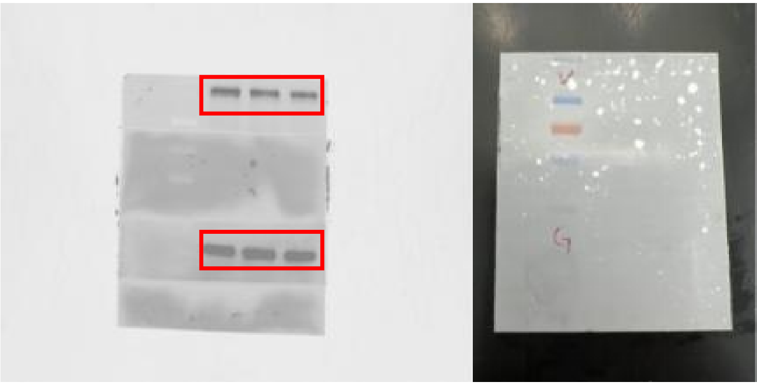

Figure2.A
Membrane VE-cadherin and
corresponding Na,K⁺ATPse

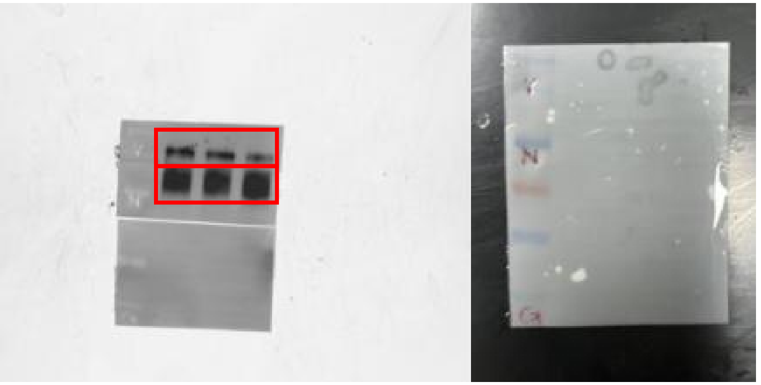

Figure2.A
Cytoplasm VE-cadherin and
corresponding GAPDH

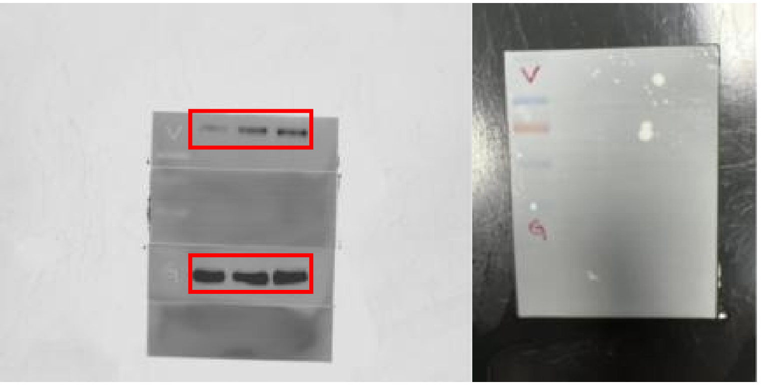

Figure2.B

ZO1, Occludin and corresponding GAPDH

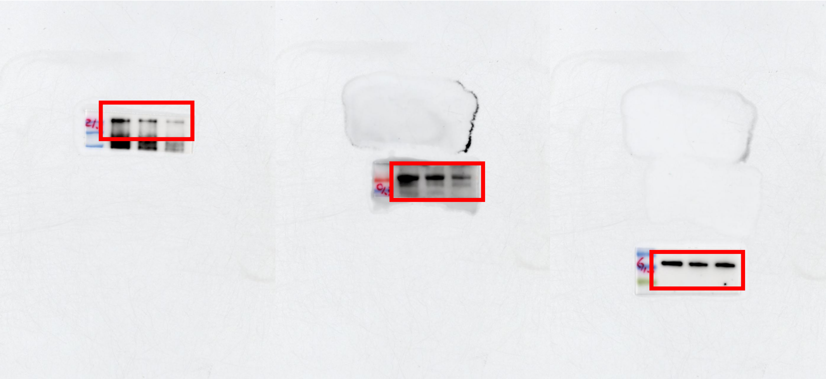

Figure2.F
Cytoplasm VE-cadherin and corresponding GAPDH

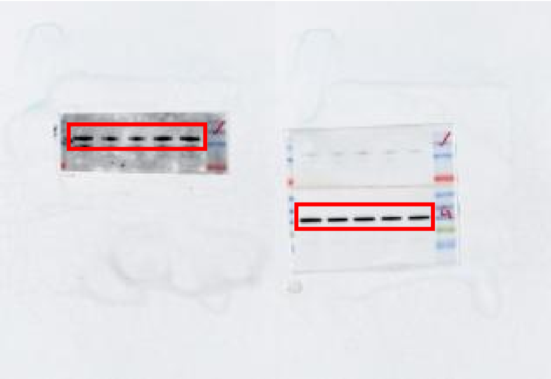

Figure 3.H
Total VE-cadherin and corresponding GAPDH

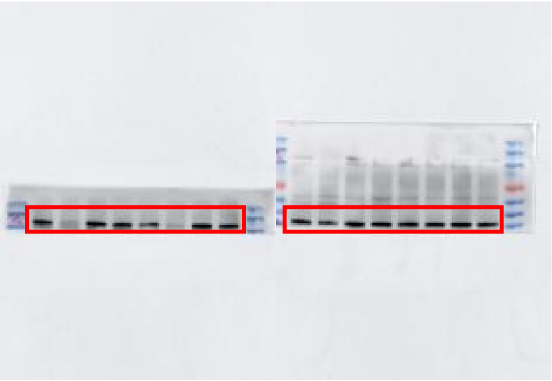

Figure 3.H
Membrane VE-cadherin and corresponding Na,K^+ATPse

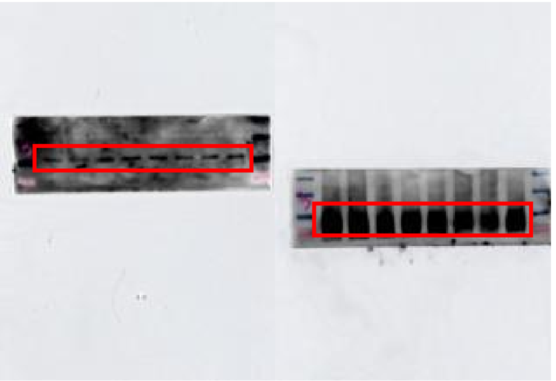

Figure 3.H
Cytoplasm VE-cadherin and corresponding GAPDH

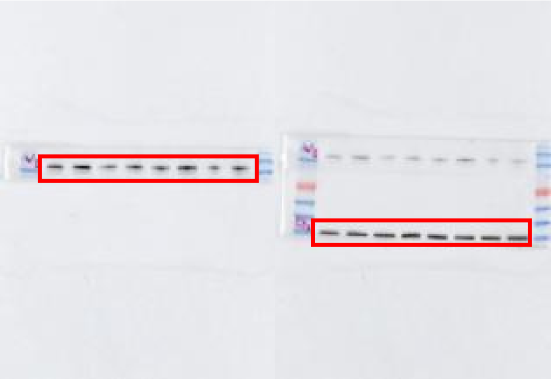

Figure 3.I

ZO1, Occludin and corresponding GAPDH

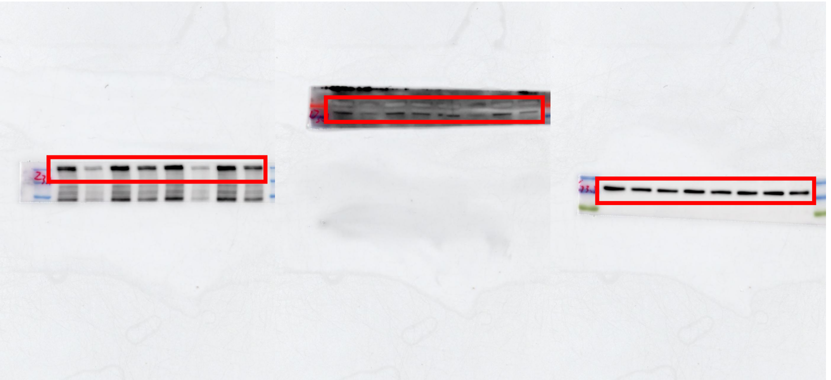

Figure 4.B
VE-cadherin and corresponding GAPDH

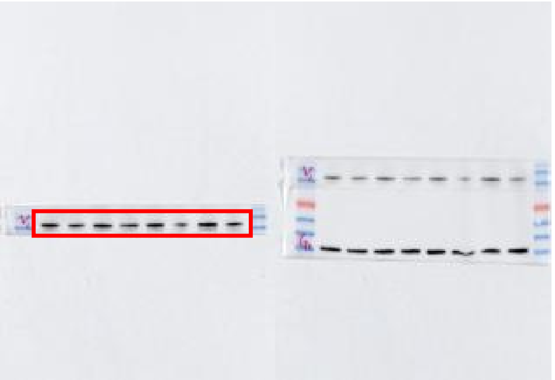

Figure 4.B

ZO1, Occludin and corresponding GAPDH

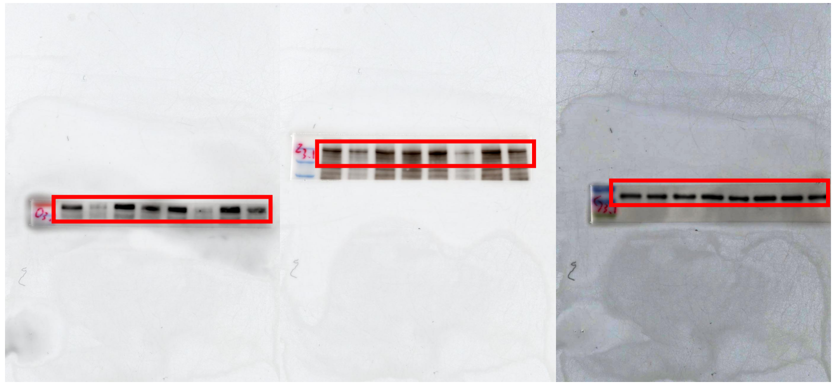

Figure 4.I

XBP1s and corresponding GAPDH

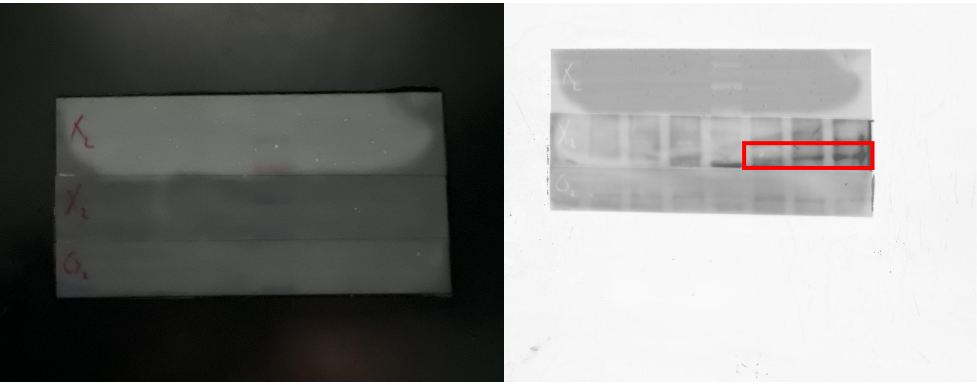

Figure 4.I

RAB7 and corresponding GAPDH

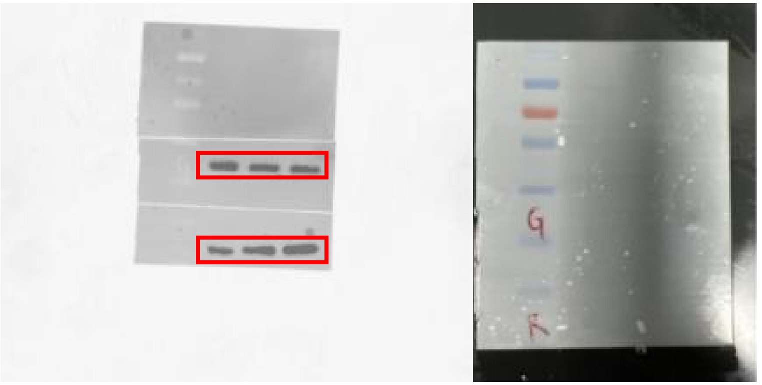

Figure 4.J

XBP1s and corresponding GAPDH

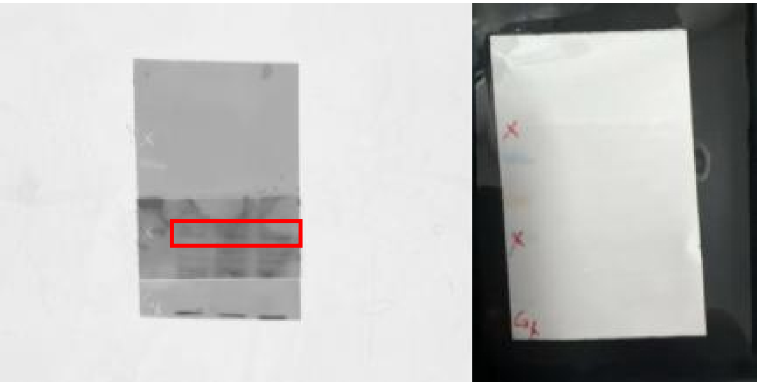

Figure 4.J

RAB7 and corresponding GAPDH

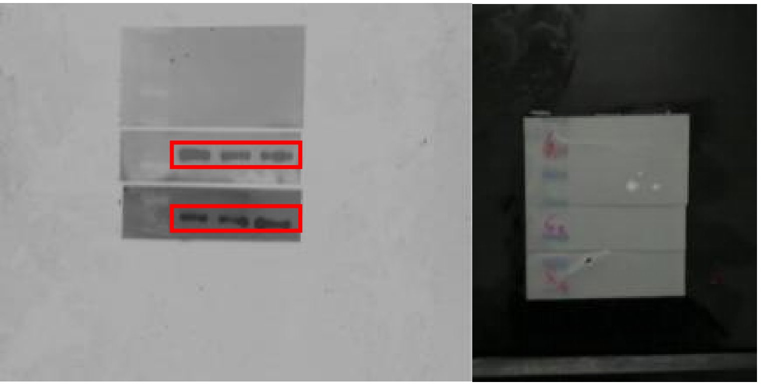

Figure 4.K

XBP1s and corresponding GAPDH

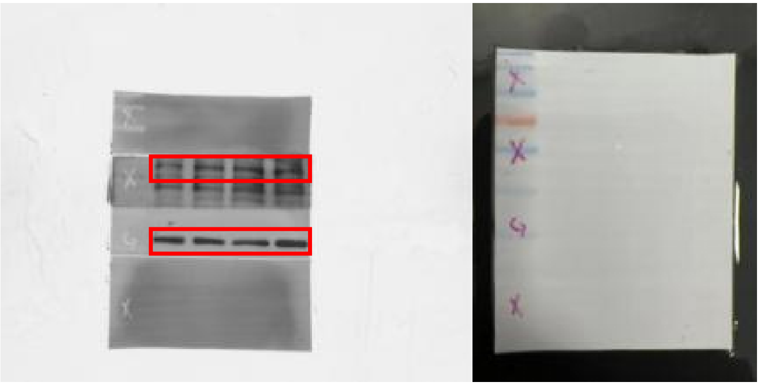

Figure 4.K

RAB7 and corresponding GAPDH

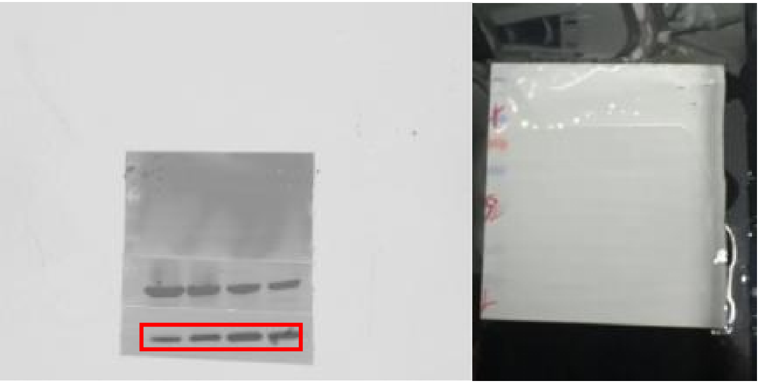

Figure 5.A

XBP1s and corresponding GAPDH

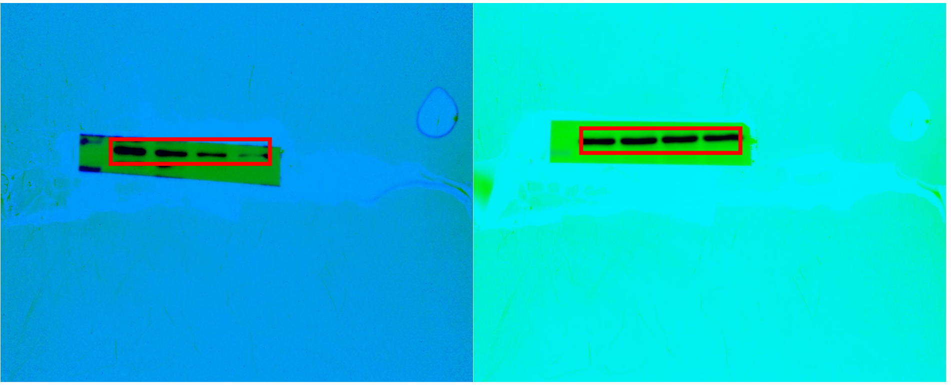

Figure 5.B

XBP1s and corresponding GAPDH

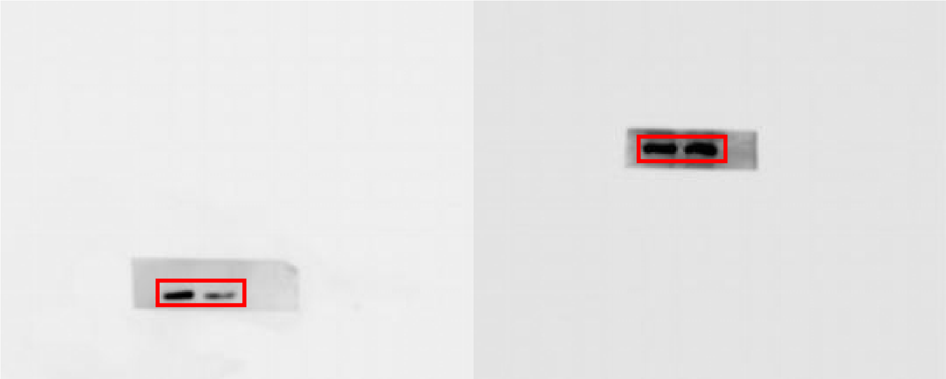

Figure 5.C
RAB7 and
corresponding GAPDH

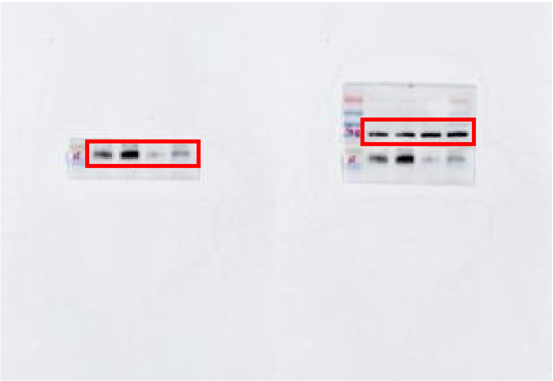

Figure 5.C

XBP1s and corresponding GAPDH

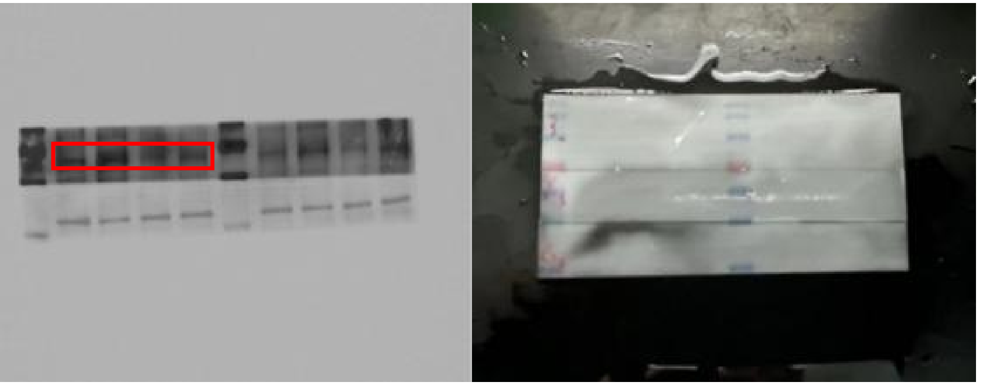

Figure 5.D
Total VE-cadherin
and corresponding GAPDH

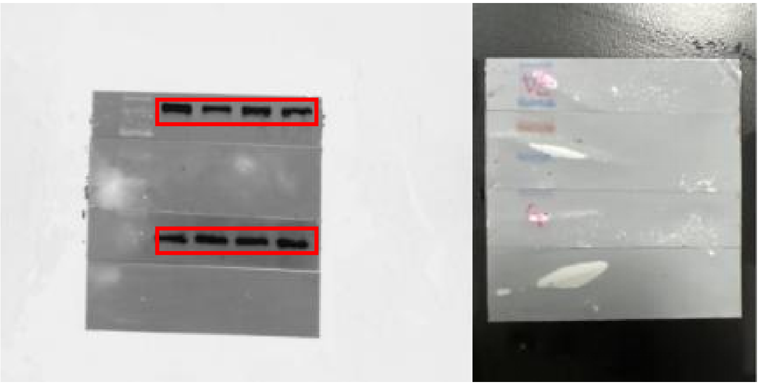

Figure 5.D
Membrane VE-cadherin
and corresponding Na,K^{+}ATPse

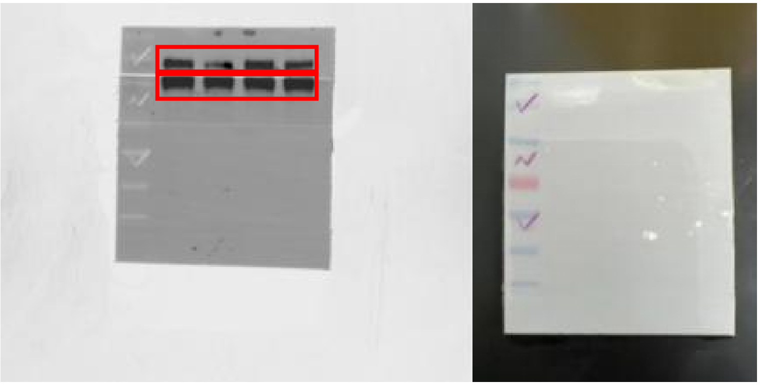

Figure 5.D

Cytoplasm VE-cadherin

and corresponding GAPDH

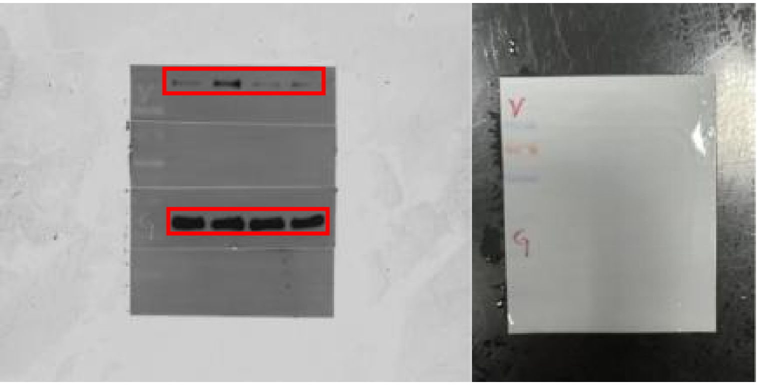

Figure 5.E

ZO1, Occludin and corresponding GAPDH

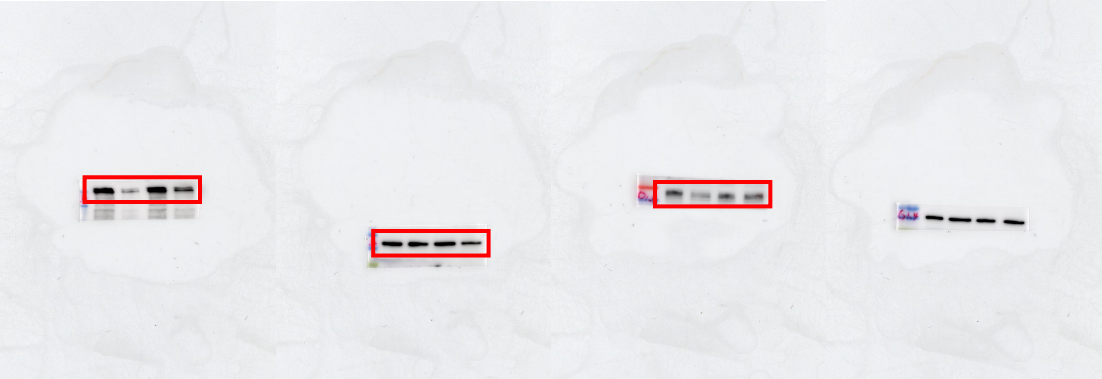

Figure 5.J

IP-RAB7 and IP-VE-cadherin

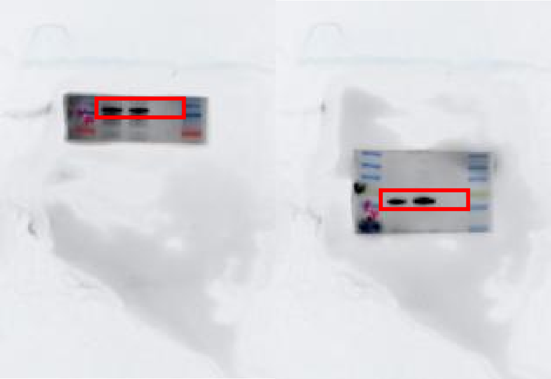

Figure 5.J

Input-RAB7 and Input-VE-cadherin

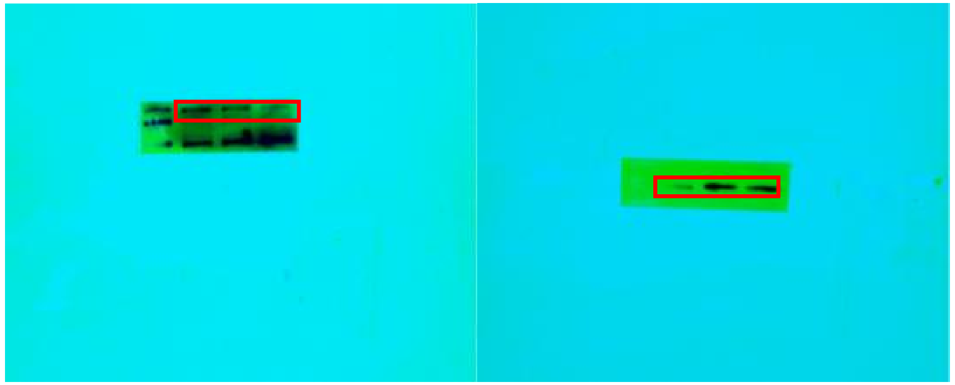

Figure 6.A

RAB7 and corresponding GAPDH

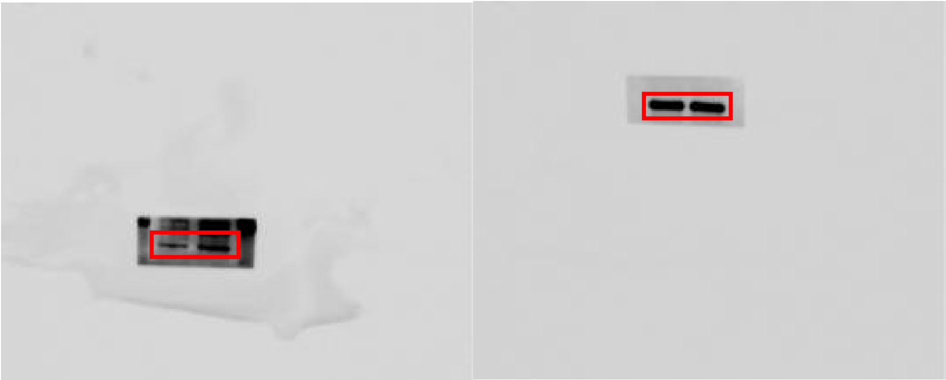

Figure 6.B

XBP1s and corresponding GAPDH

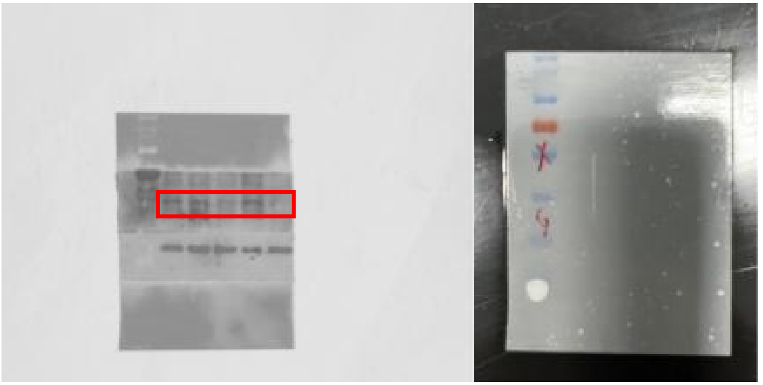

Figure 6.B

RAB7 and corresponding GAPDH

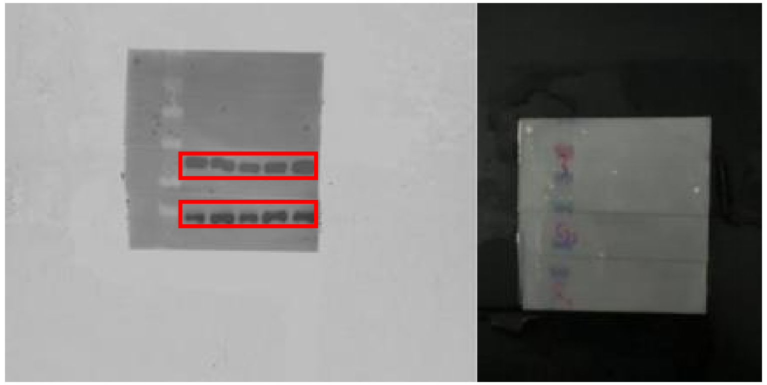

Figure 6.C
Total VE-cadherin
and corresponding GAPDH

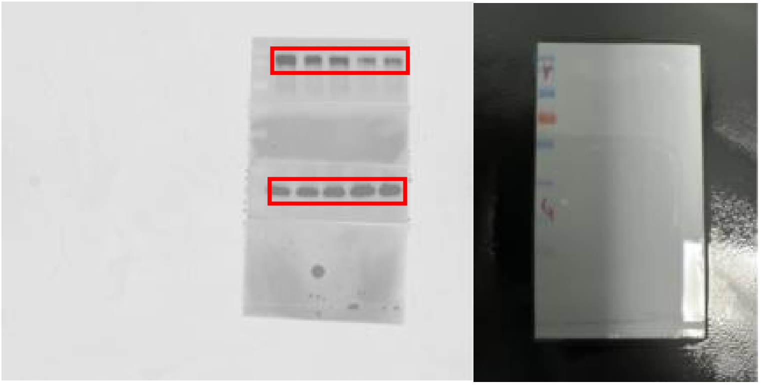

Figure 6.C
Membrane VE-cadherin and
corresponding Na,K$^+$ATPse

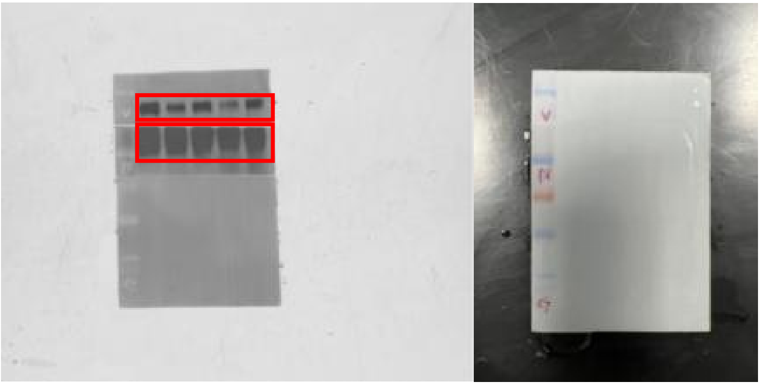

Figure 6.C
Cytoplasm VE-cadherin
and corresponding GAPDH

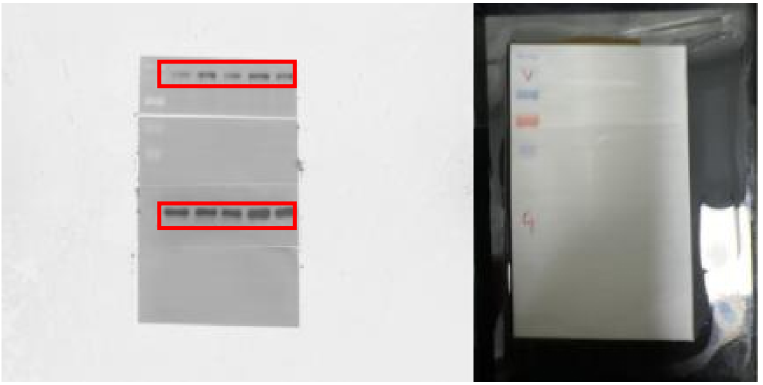

Figure 6.D

ZO1, Occludin and corresponding GAPDH

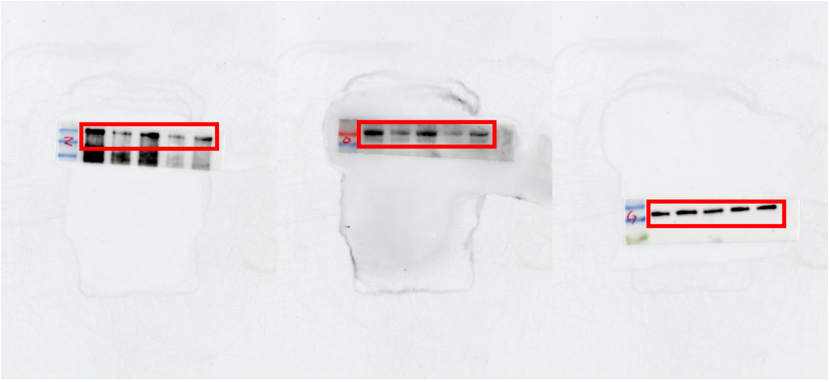

Figure 7.B

VE-cadherin and corresponding GAPDH

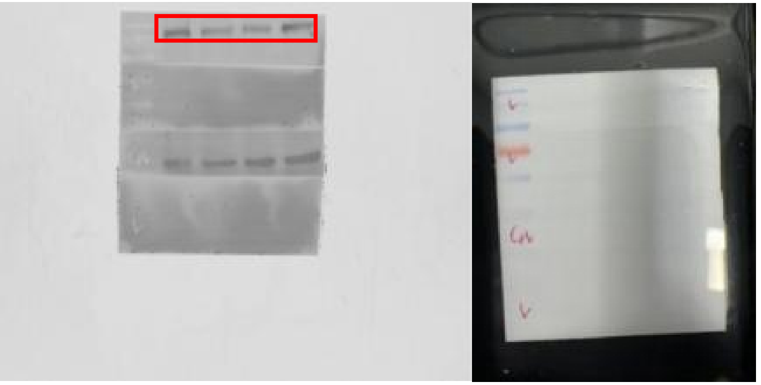

Figure 7.B

XBP1s and corresponding GAPDH

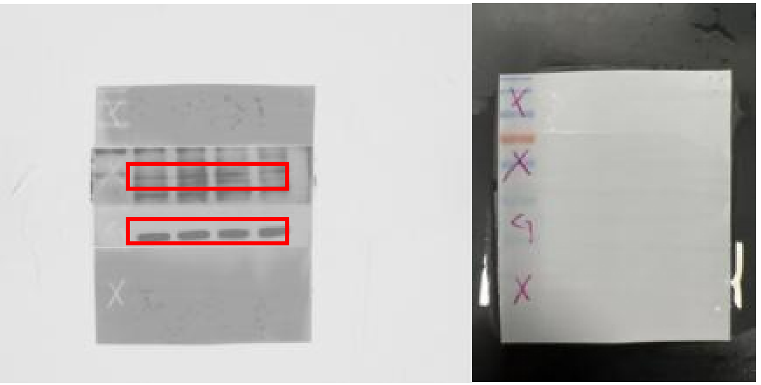

Figure 7.B

ZO1, Occludin and corresponding GAPDH

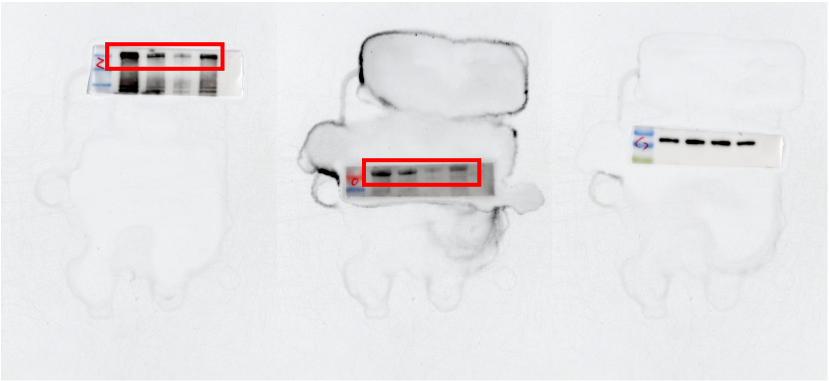

Figure 7.B

RAB7 and corresponding GAPDH

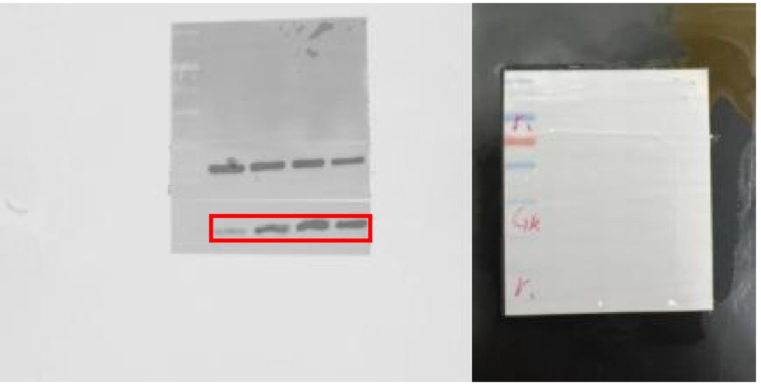

Figure S1.B
Apolipoprotein A1 in both groups of EVs

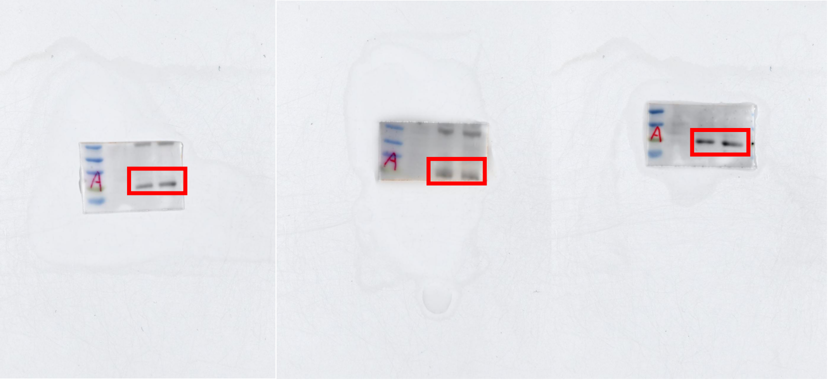

Figure S2.A
Na,K^+ATPse and GAPDH

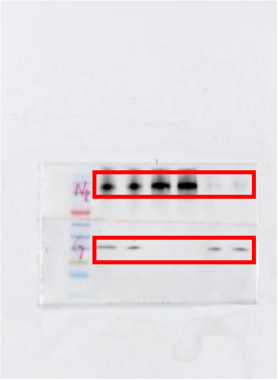

Figure S2.B
VE-cadherin and corresponding Tubulin

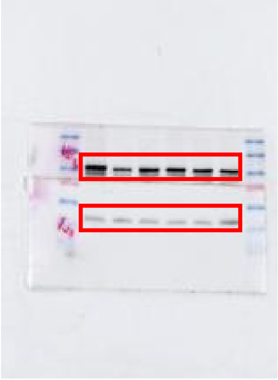

Figure S3.B
EVs markers
CD63

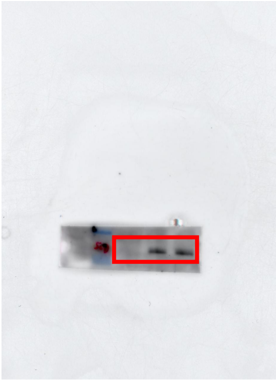

Figure S3.B
EVs markers
Calnexin

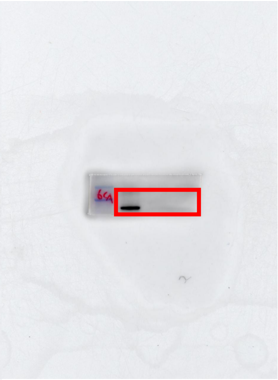

Figure S3.E
VE-Cadherin, ZO1, Occludin and corresponding GAPDH

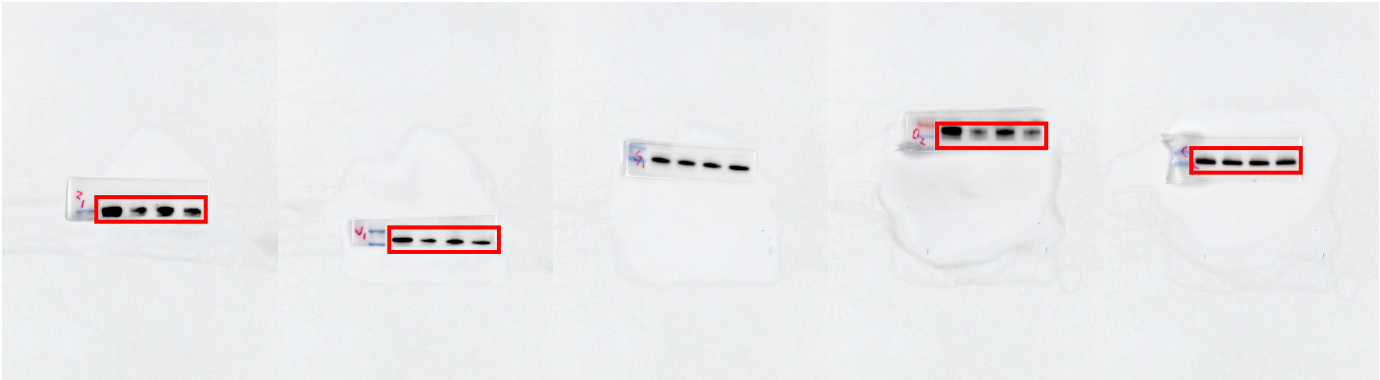

Figure S4.B
VE-cadherin and corresponding GAPDH

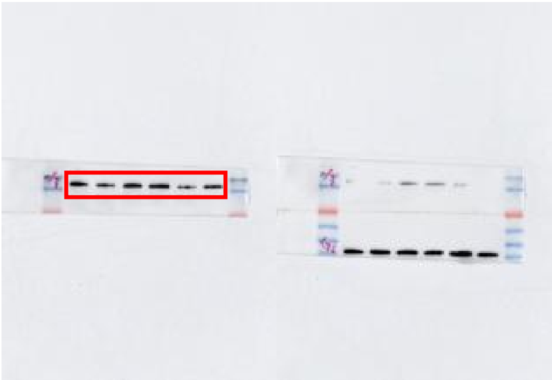

Figure S4.B

XBP1s and corresponding GAPDH

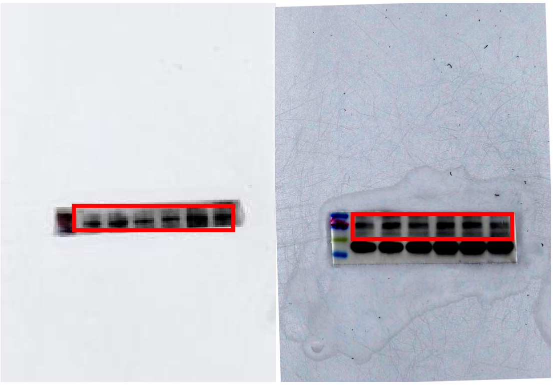

Figure S4.B

RAB7 and corresponding GAPDH

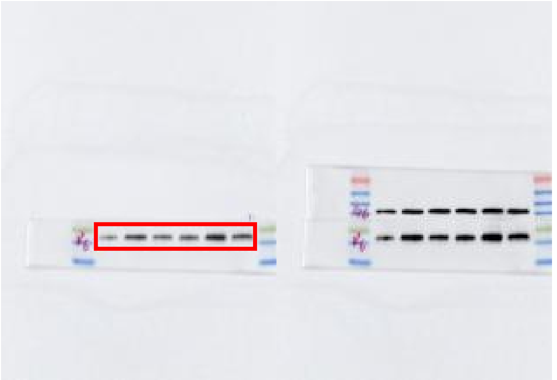

Figure S4.B

ZO1, Occludin and corresponding GAPDH

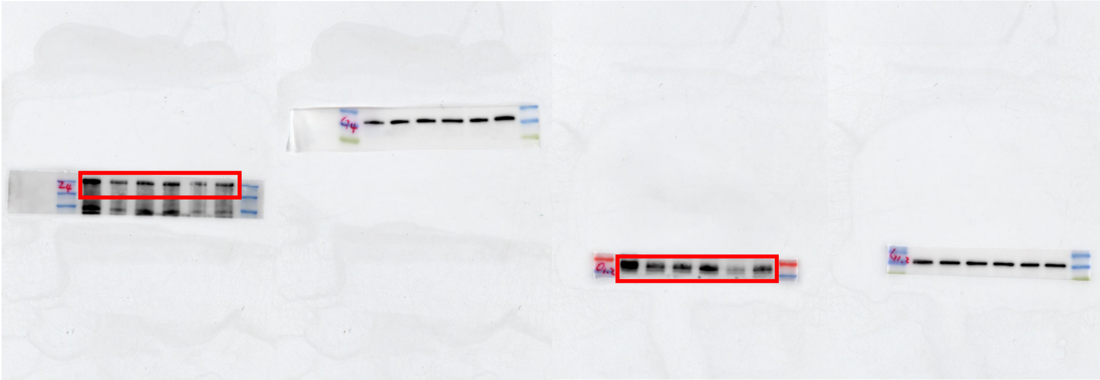

Supplement: Supplementary file 7 — Full length western blots [file 41420_2025_2499_MOESM7_ESM.pdf]
